# Elevation of fatty acid desaturase 2 in esophageal adenocarcinoma increases polyunsaturated lipids and may exacerbate bile acid‐induced DNA damage

**DOI:** 10.1002/ctm2.810

**Published:** 2022-05-12

**Authors:** Jeffrey Molendijk, Cathryn M. Kolka, Henry Cairns, Sandra Brosda, Ahmed Mohamed, Alok K. Shah, Ian Brown, Mark P. Hodson, Thomas Hennessy, Guanghao Liu, Thomas Stoll, Renee S. Richards, Michael Gartside, Kalpana Patel, Nicholas J. Clemons, Wayne A. Phillips, Andrew Barbour, Johan A. Westerhuis, Michelle M. Hill

**Affiliations:** ^1^ The University of Queensland Diamantina Institute, Faculty of Medicine The University of Queensland Woolloongabba Australia; ^2^ Precision and Systems Biomedicine Laboratory QIMR Berghofer Medical Research Institute Herston Australia; ^3^ Envoi Pathology Herston Australia; ^4^ School of Pharmacy The University of Queensland Woolloongabba Australia; ^5^ Agilent Technologies Mulgrave Australia; ^6^ Division of Cancer Research Peter MacCallum Cancer Centre Melbourne Australia; ^7^ Sir Peter MacCallum Department of Oncology The University of Melbourne Parkville Australia; ^8^ Swammerdam Institute for Life Sciences University of Amsterdam Amsterdam The Netherlands

**Keywords:** Barrett's esophagus, esophageal adenocarcinoma, FADS2, lipid desaturation, lipid metabolism

## Abstract

**Background:**

The risk of esophageal adenocarcinoma (EAC) is associated with gastro‐esophageal reflux disease (GERD) and obesity. Lipid metabolism‐targeted therapies decrease the risk of progressing from Barrett's esophagus (BE) to EAC, but the precise lipid metabolic changes and their roles in genotoxicity during EAC development are yet to be established.

**Methods:**

Esophageal biopsies from the normal epithelium (NE), BE, and EAC, were analyzed using concurrent lipidomics and proteomics (*n* = 30) followed by orthogonal validation on independent samples using RNAseq transcriptomics (*n* = 22) and immunohistochemistry (IHC, *n* = 80). The EAC cell line FLO‐1 was treated with FADS2 selective inhibitor SC26196, and/or bile acid cocktail, followed by immunofluorescence staining for γH2AX.

**Results:**

Metabolism‐focused Reactome analysis of the proteomics data revealed enrichment of fatty acid metabolism, ketone body metabolism, and biosynthesis of specialized pro‐resolving mediators in EAC pathogenesis. Lipidomics revealed progressive alterations (NE‐BE‐EAC) in glycerophospholipid synthesis with decreasing triglycerides and increasing phosphatidylcholine and phosphatidylethanolamine, and sphingolipid synthesis with decreasing dihydroceramide and increasing ceramides. Furthermore, a progressive increase in lipids with C20 fatty acids and polyunsaturated lipids with ≥4 double bonds were also observed. Integration with transcriptome data identified candidate enzymes for IHC validation: Δ4‐Desaturase, Sphingolipid 1 (DEGS1) which desaturates dihydroceramide to ceramide, and Δ5 and Δ6‐Desaturases (fatty acid desaturases, FADS1 and FADS2), responsible for polyunsaturation. All three enzymes showed significant increases from BE through dysplasia to EAC, but transcript levels of DEGS1 were decreased suggesting post‐translational regulation. Finally, the FADS2 selective inhibitor SC26196 significantly reduced polyunsaturated lipids with three and four double bonds and reduced bile acid‐induced DNA double‐strand breaks in FLO‐1 cells in vitro.

**Conclusions:**

Integrated multiomics revealed sphingolipid and phospholipid metabolism rewiring during EAC development. FADS2 inhibition and reduction of the high polyunsaturated lipids effectively protected EAC cells from bile acid‐induced DNA damage in vitro, potentially through reduced lipid peroxidation.

## Graphical abstract

Multiomic techniques were applied to the normal esophagus, Barrett's esophagus, and esophageal adenocarcinoma (EAC) tissues to investigate lipid metabolism. Polyunsaturated lipids, desaturase pathways, and antioxidant processes were upregulated as the disease progresses. Blocking fatty acid desaturase 2 protects an EAC line from bile acid‐induced DNA damage.

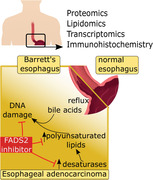

## 1 INTRODUCTION

The co‐incidental rise of esophageal adenocarcinoma (EAC) and obesity over the past decades has prompted investigations into obesity‐associated carcinogenic mechanisms in EAC.^1^ EAC carcinogenesis is thought to be driven by oxidative stress and inflammation caused by chronic gastro‐esophageal reflux,^2^ with Barrett's esophagus (BE) being the only known precursor condition to EAC. Obesity increases the risk of gastro‐esophageal reflux disease (GERD), BE, and EAC, with diverse mechanisms proposed, including defective lower esophageal sphincter function, systemic inflammation, metabolism dysregulation, and metabolic syndrome.^1^ Obesity‐associated metabolic deregulation contributes to the cancer hallmark ‘deregulating cellular energetics’ through increased fatty acid synthesis. Elevated levels of enzymes responsible for lipid elongation (ELOVL), monounsaturation (Stearoyl‐CoA Desaturases [SCD]), and polyunsaturation (fatty acid desaturases [FADS]) have been reported in other cancers.^3^, ^4^

In addition to deregulation of cellular energetics, higher levels of polyunsaturated lipids also present more targets for attack by reactive oxygen species (ROS). Bile acids in the refluxate induce ROS in esophageal cells via diverse pathways such as the NADPH oxidases NOX1 and NOX2, and nitric oxide synthases.^5^, ^6^, ^7^ Together with dysfunction in detoxifying antioxidant enzymes that have been characterized in cancers, the unchecked ROS can cause peroxidation of the double bonds in polyunsaturated lipids, generating genotoxic reactive aldehyde byproducts.^8^ Oxidative DNA damage along with evasion of apoptosis/ferroptosis leads to extensive genomic mutations in BE and EAC.^9^ Evidence for the accumulation of genotoxic lipid aldehydes in EAC was recently reported by Antonowicz et al..^10^ However, there has only been a single study reporting lipidomics profiles for BE and EAC tissues.^11^ Desorption electrospray ionization‐mass spectrometry imaging identified a progressive increase in polyunsaturated long‐chain glycerophospholipids and the lipid class phosphatidylglycerol (PG) in the stages of EAC development, with a significant increase in glycerophospholipids with four double bonds and 38 total acyl chains, and a parallel decrease in monounsaturated glycerophospholipids and 34 total acyl chains.^11^ PG biosynthetic genes LPGAT and PGS1 were found to be increased in EAC compared to the healthy esophagus in archived transcriptome data, while immunohistochemical analysis identified elevated expression of enzymes involved in the fatty acid synthesis and monounsaturation (SCD).^11^ The Δ9‐desaturase SCD1, which introduces a single double bond between the 9th and 10th carbons from the fatty acid carboxylate terminus of long‐chain saturated acyl‐CoA has been explored as a therapeutic target in a range of metabolic diseases, skin disorders and cancers.^12^ However, metabolic plasticity in fatty acid desaturation, exemplified by the desaturation of palmitate to sapienate, was recently highlighted as a means of evading SCD‐targeted therapies in cancer cell lines.^13^, ^14^ Therefore, comprehensive knowledge of the lipid metabolic landscapes of BE and EAC is a prerequisite to support the design of chemopreventative or therapeutic strategies for this increasingly prevalent cancer of poor prognosis.

To clarify the changes in the lipid metabolic landscape during the development of EAC, we conducted exploratory tissue proteomic, lipidomic, and transcriptomic analyses on a limited set of esophageal biopsies from BE and EAC patients. Integrative analysis of these datasets identified polyunsaturated fatty acid and ceramide (Cer) biosynthetic enzymes as potential mediators of lipid metabolic rewiring in EAC progression. The protein expression of fatty acid and sphingolipid desaturates (FADS1, FADS2, DEGS1) were validated by immunohistochemistry (IHC) in an independent set of tissue samples including stages of dysplasia. Finally, the function of FADS in polyunsaturated lipid biosynthesis was evaluated using inhibitor studies in the EAC cell line, FLO‐1.

## 2 RESULTS

### 2.1 Changes to lipids and proteins involved in lipid metabolism during EAC progression

**FIGURE 1:**
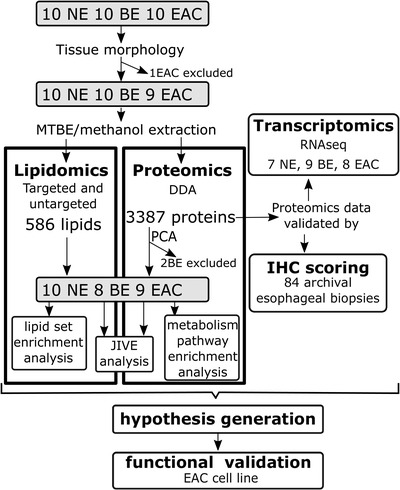
**Workflow for patient sample multiomic analyses and immunohistochemistry validation**. Three different cohorts were used for multiomics analyses and validation of selected proteins. Concurrent lipidomics and proteomics were conducted on 10 biopsies each from the normal epithelium (NE), Barrett's esophagus (BE), and esophageal adenocarcinoma (EAC). Data from three samples were excluded from further analyses after quality control by tissue morphology or principal component analysis (PCA, Figure S1). The second set of biopsies was used for RNAseq transcriptomic analysis and used to support pathway interpretation. Finally, tissue expression levels of selected proteins in lipid metabolism were validated by immunohistochemistry conducted on a tissue microarray generated from 84 archival esophageal biopsies. MTBE, Methyl tert‐butyl ether; DDA, data‐dependent acquisition; IHC, immunohistochemistry; JIVE, Joint and Individual Variation Explained.

Figure 1 depicts the workflow in this study, which began with exploratory proteomics and lipidomics on esophageal tissue biopsies, corroboration, and validation using transcriptomics and IHC, followed by hypothesis generation and functional evaluation. Ten esophageal biopsy samples collected from each normal esophagus (NE), BE, and EAC were chosen for concurrent global proteomics and lipidomics analyses. Tissue morphology was confirmed by histology of an adjacent biopsy, which excluded one EAC sample as histology indicated squamous cell carcinoma instead of EAC (Figure 1). Lipids and proteins were simultaneously extracted and subjected to untargeted data‐dependent acquisition proteomics and previously published targeted and untargeted lipidomics methods.^15^ The targeted lipidomics measured 228 lipid species (Cer, DHCer, ether‐PC, ether‐PE, LPC, LPE, PC, PE, PG, PI, PS, SM), while the untargeted lipidomics data were mapped to 366 lipids (CE, Cer, Cer‐AS, Cer‐BS, DG, DGDG, DHCer, Ether‐LPC, ether‐LPE, ether‐PC, ether‐PE, FAHFA, HBMP, HexCer‐AP, HexCer‐NS, LPA, LPC, LPE, LPI, LPS, MGDG, OxLPC, PA, PC, PE, PEtOH, PG, PI, PS, SM, So, TG) (Abbreviations in Table S1, Lipidomic data with differential analysis Table S2). Proteomics and lipidomics data were separately processed through quality control, normalization, and imputation as described in Methods, resulting in final datasets of 586 lipids and 3387 proteins. Principal component analysis (PCA) of the lipidomics and proteomics datasets shows clustering of samples according to tissue type, except for 2 BE samples that clustered with NE samples (Figure S1A,B). As tissue type (NE, BE, or EAC) was determined based on biopsies adjacent to the ones used for lipidomics/proteomics, it is possible that two adjacent biopsies from the same patient show a different phenotype. In support of this explanation, proteomics data of NE and BE tissues identified two BE samples (B6, B7) lacking known markers for intestinal metaplasia (Figure S1C). With the verified sampling error, these two samples were excluded from subsequent lipidomic and proteomic analyses. Therefore, the final cohort comprised 10 NE, eight BE, and nine EAC (Figure 1). Participant characteristics are shown in Table S3, however dietary and GERD information were not available. There were no significant differences in age, sex, body mass index/body weight, smoking, or alcohol consumption between the groups (Table S3).

**FIGURE 2:**
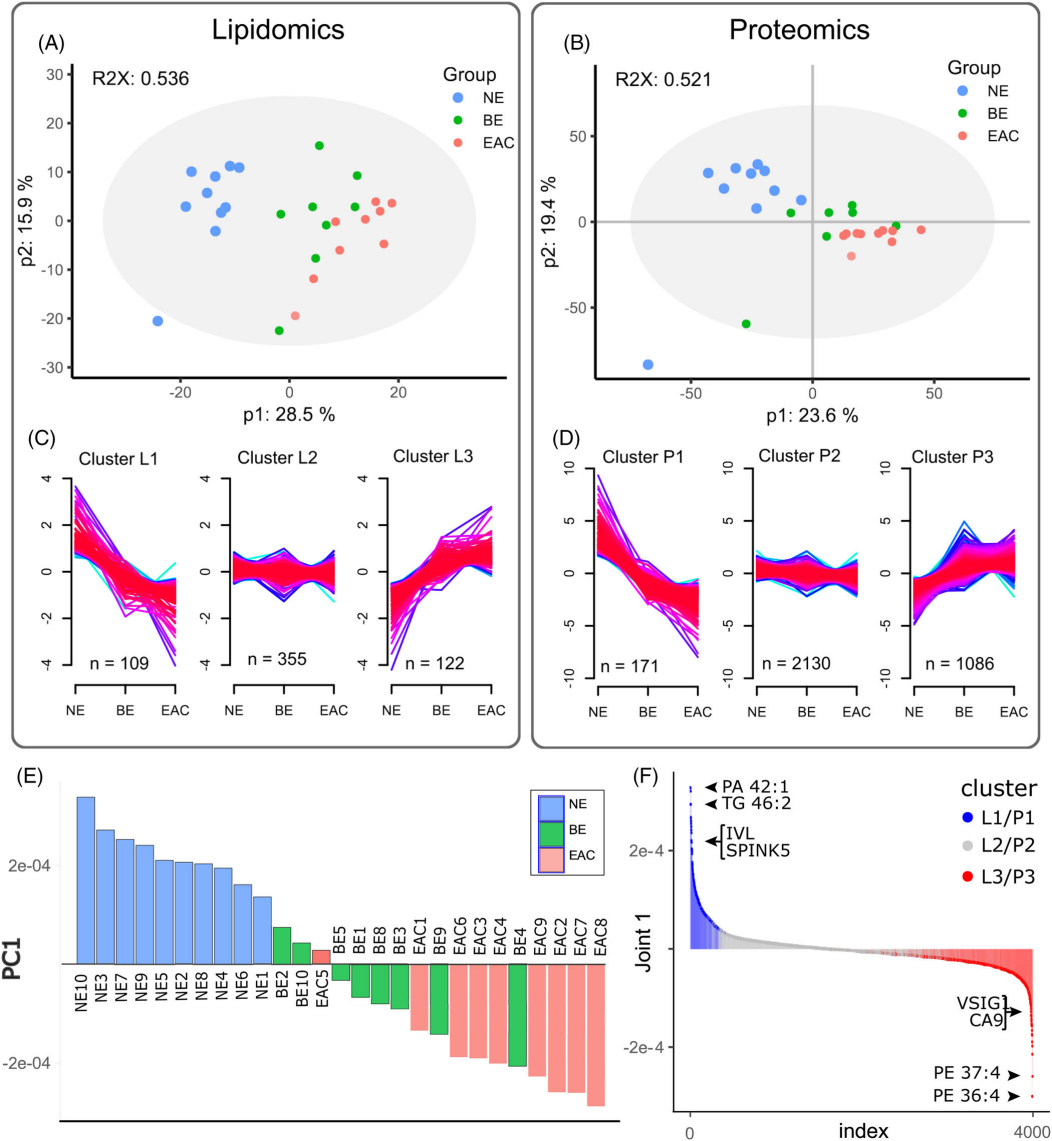
**Overview of omics data** . Principal component analysis (PCA) of patient biopsy lipidomics data (*n* = 586 lipids) (A) and proteomics data (*n* = 3387 proteins) (B) after exclusions revealed distinct clusters. Fuzzy C‐means clustering was used to divide the lipid and protein datasets into three clusters; L1–L3 for lipids (C) and P1–P3 for proteins (D), demonstrating a progressive change from the normal esophagus (NE, n = 10) through Barrett's esophagus (BE, *n* = 8) to esophageal adenocarcinoma (EAC, *n* = 9). (E) Joint and Individual Variation Explained (JIVE) analysis of the lipidomics and proteomics data revealed a single significant component (PC1) which divides the samples into the progression continuum of NE, BE, EAC shown in a bar graph. The proteins and lipids in PC1 are shown in Table S3, and the top JIVE components are shown in (F).

Fuzzy C‐means clustering^16^ was used to further characterize the altered lipids (Figure 2A) and proteins (Figure 2B), revealing a similar number of measured lipid species were up and down‐regulated (122 and 106, respectively, Figure 2C) d in a step‐wise manner, while 1086 proteins were progressively upregulated but only 171 proteins were progressively downregulated (Figure 2D), in the development of EAC. To integrate lipidomics and proteomics data, we applied Joint and Individual Variation Explained (JIVE)^17^, ^18^ decomposition analysis, which revealed joint variation between the lipidomics and proteomics datasets according to disease stage, as well as variation unique to the separate datasets (Figure S2). The joint variation accounts for over 30% of all variation in both lipidomic and proteomic data, supporting a change in lipid pathways in EAC (Figure S2B). PCA of the joint variation indicates that the first joint component highlights a progression of esophageal lipidome and proteome from NE, BE to EAC (Figure 2E). Loadings from this analysis are presented in Table S4 with top lipids and proteins indicated in Figure 2F. Lipid species, particularly the reduction of triglycerides dominates PC1, while phospholipids with a large number of double bonds are increased in EAC. The top proteins have known roles in esophageal tissue organization or cancer: IVL is a classic marker of cornification characteristic of normal epithelium^19^ and SPINK5 is associated with esophageal cancer suppression.^20^ Conversely, CA9,^21^, ^22^ has been reported to be elevated in BE/EAC and hypoxia in cancer.^23^

**FIGURE 3:**
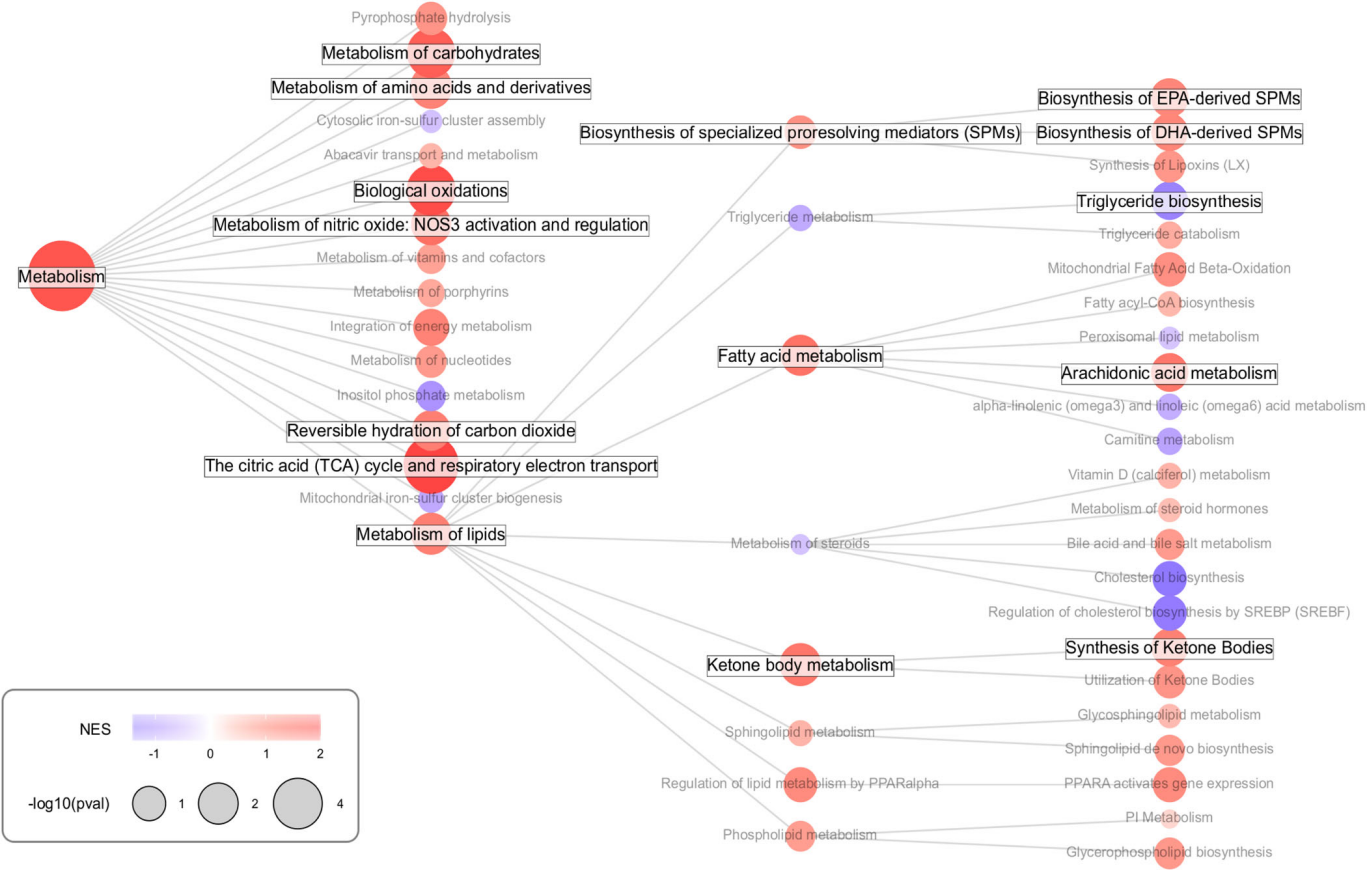
**Metabolism‐focussed pathway enrichment analysis of the tissue proteomics data using Reactome**. Larger circles indicate more significant enrichment, color indicates normalized enrichment score (NES). Names of significant (*p* < .05) gene sets are highlighted in bold

To focus on the metabolism changes during EAC development, we applied enrichment analysis to only the metabolic and lipid metabolic pathways in the Reactome (Figure 3). Positive enrichment of lipid, carbohydrate, amino acid, and nitric oxide metabolism, as well as biological oxidations in the citric acid (TCA) cycle, were found. Significantly altered pathways downstream of “metabolism of lipids” include the metabolism of fatty acids, ketone bodies, and specialized pro‐resolving mediators (SPMs) (positive enrichment) as well as the biosynthesis of triglycerides (negative enrichment). Ketone bodies and SPMs were not measured in our lipidomics dataset, as the lipidomics analyses focused on phospholipids, sphingolipids, and triglycerides.

### 2.2 Lipid class, unsaturation, and chain length changes occur in BE and EAC

**FIGURE 4:**
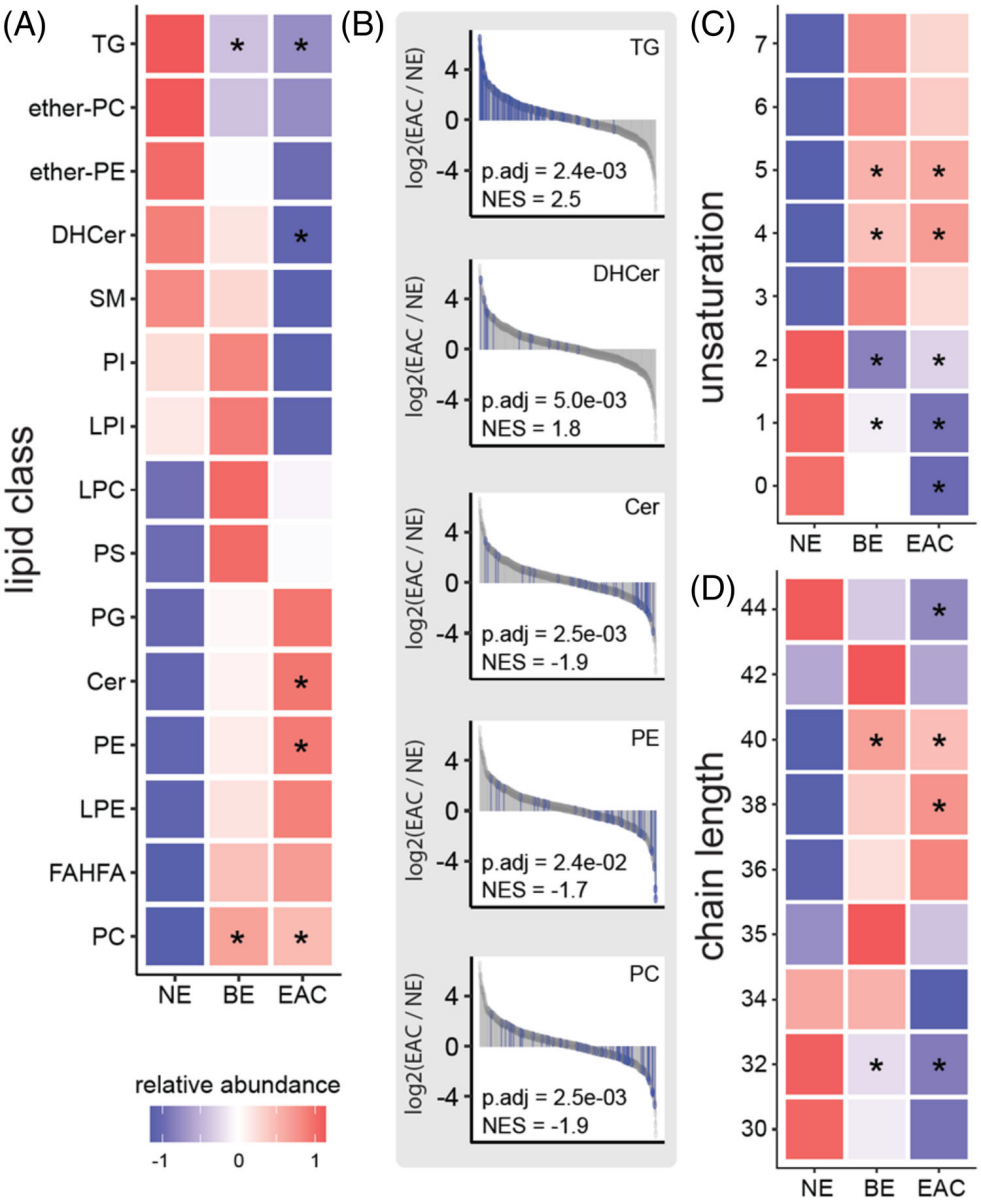
**Overview of lipidomics data using lipid set enrichment analysis**. (A) Lipid class enrichment comparing normal esophagus (NE, *n* = 10) with Barrett's esophagus (BE, *n* = 8) and esophageal adenocarcinoma (EAC, *n* = 9). Lipid set enrichment analysis was performed on log2 fold‐changes and data was centered and scaled prior to visualization. (B) Individual results for lipid classes altered in EAC compared to NE. Lipids belonging to the corresponding lipid class are highlighted in blue. (C) Unsaturation enrichment for lipids with two fatty acids (including Cer‐NDS, Cer‐NS, PC, PG, ether‐PC, ether‐PE, PS, SM FAHFA, PE, PI) demonstrates an increase in lipids with unsaturation of 4 and 5, and a decrease in saturated lipids (D) Chain length analysis for lipids with two fatty acids. NES: normalized enrichment score. *p.adj < .05

As an overview, the progressive lipidome alterations were analyzed using lipid set enrichment analysis,^24^ and the lipid classes were visualized by heatmap (Figure 4A) and waterfall plots (Figure 4B). Reflecting the Reactome pathway enrichment (Figure 3), a significant progressive decrease in triacylglycerols was observed. The lipidomics data further revealed a significant, progressive increase in the glycerophospholipids phosphatidylethanolamines (PE) and phosphatidylcholines (PC) in BE and EAC. The lipidomics data also revealed specific alterations in the sphingolipids, ceramide, and dihydroceramide (DHCer) (Figure 4A, B).

Next, we investigated total fatty acid chain length and level of unsaturation (number of double bonds) as these properties impact membrane/cellular lipid function.^25^ For this analysis, lipids with one (Figure S3A,B), two (Figure 4C,D), or three (Figure S3C,D) fatty acids were separately evaluated to distinguish the non‐membrane triglycerides (three fatty acids) from membrane lipids (including lysophospholipids, which have one fatty acid chain, and phospholipids and sphingolipids with two chains). The enrichment of arachidonic acid (C20:4) metabolism‐related proteins (Figure 3) is supported by a significant increase in lipids with 4 unsaturated bonds (Figure S3A) and a chain length of 20 (Figure S3B). These lipids may be incorporated into larger lipids, and therefore be associated with the significant increase in C38 and C40 lipids, which likely contain a C20 lipid. Strikingly, regardless of the number of fatty acids, all lipids with four and five double bonds were significantly increased in EAC. Conversely, there was a decrease in lipids with zero, one, or two unsaturated bonds in BE and EAC, regardless of the number of fatty acids (Figure 4C and Figure S3).

### 2.3 Integrated transcriptomic and proteomic analysis implicate lipid desaturation in EAC

**FIGURE 5:**
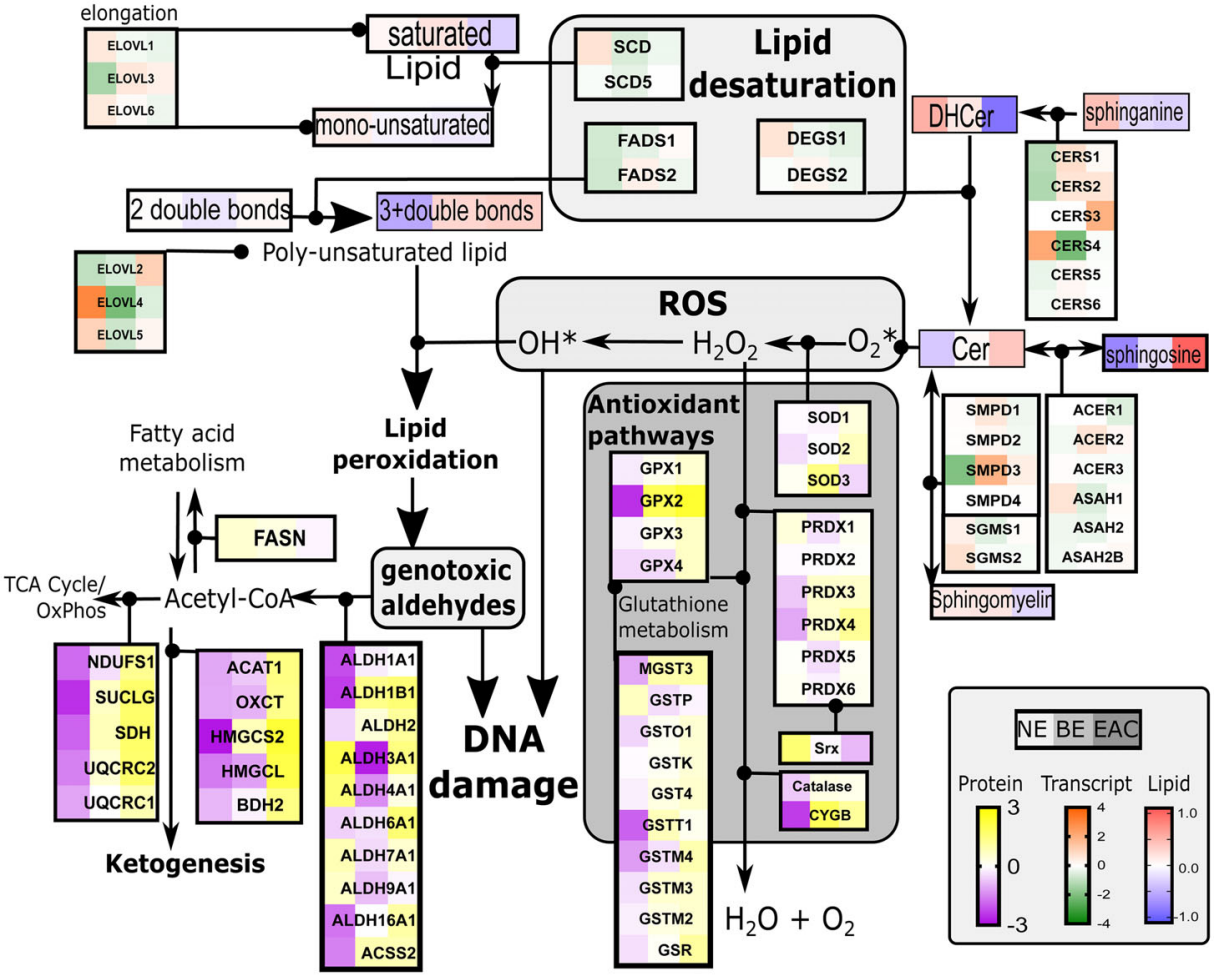
**Integrated multiomic data highlight altered lipid desaturation and antioxidant pathways during progression from the normal esophagus (NE) to Barrett's esophagus (BE) to esophageal adenocarcinoma (EAC)**. The proteomic, lipidomic, and transcriptomic data from independent cohorts are shown with heatmaps representing the median of the mean‐centered data

Prior to selecting target lipid metabolic enzymes for wet‐lab validation, we next evaluated the enzymes associated with elongation and desaturation during EAC development. As the shotgun proteomics data did not include lipid elongation (ELOVL) or desaturation enzymes (SCD, FADS, and DEGS), possibly due to low protein abundance, we sought to establish the relative transcript levels of these enzymes using the transcriptome data from an independent cohort of 24 samples (seven NE, nine BE, and eight EAC). An integrated pathway map of lipid elongation, desaturation, lipid oxidation, antioxidant, and ketogenesis pathways was developed to visualize lipid class abundance, transcript expression, and protein abundance changes across the stages of NE, BE, and EAC (Figure 5).

With the exception of ELOVL2, transcripts for monounsaturated lipid elongation enzymes ELOVL1, ELOVL6, and desaturases SCD, SCD5 tended to decrease with disease progression (Figure 5). On the other hand, transcripts for elongases that modify polyunsaturated lipids, ELOVL2 and ELOVL5, and FADS1 which adds the 4th double bond to a polyunsaturated fatty acid, were elevated in EAC (Figure 5). Furthermore, the observed increase in Cer and decrease in DHCer may be due to the down‐regulation in the transcripts of the dihydroceramide desaturase (DEGS) DEGS1 (Figure 5).

The increased polyunsaturated lipids may drive the observed elevation in ketogenesis and aldehyde dehydrogenase (ALDH) enzymes, and increase antioxidant pathways at the protein (Figure 5 and Figure S4) and transcript levels (Figure S5). Many of these antioxidant enzymes are elevated in other cancers and have been proposed and investigated as therapeutic targets.^26^ We have found that ALDHs are variably affected in BE and EAC patients compared to NE (protein: Figure S4L–Q, transcript: Figure S5 J–M). Consistent with a recent study,^10^ ALDH4A1 and ALDH9A1 proteins were down‐regulated in BE and EAC relative to NE, yet ALDH1A1, ALDH1B1, ALDH16A1 were elevated (Figures S4 and S5). ALDH3A1 appeared to have a transient decrease in BE (Figure S4), before a return to healthy levels in EAC, and a previous study confirmed a decrease in ALDH3A1 in EAC by immunostaining,^10^ suggesting that these changes may not necessarily be linear throughout disease stages, and changes are protein‐specific.

### 2.4 Tissue IHC validation

**FIGURE 6:**
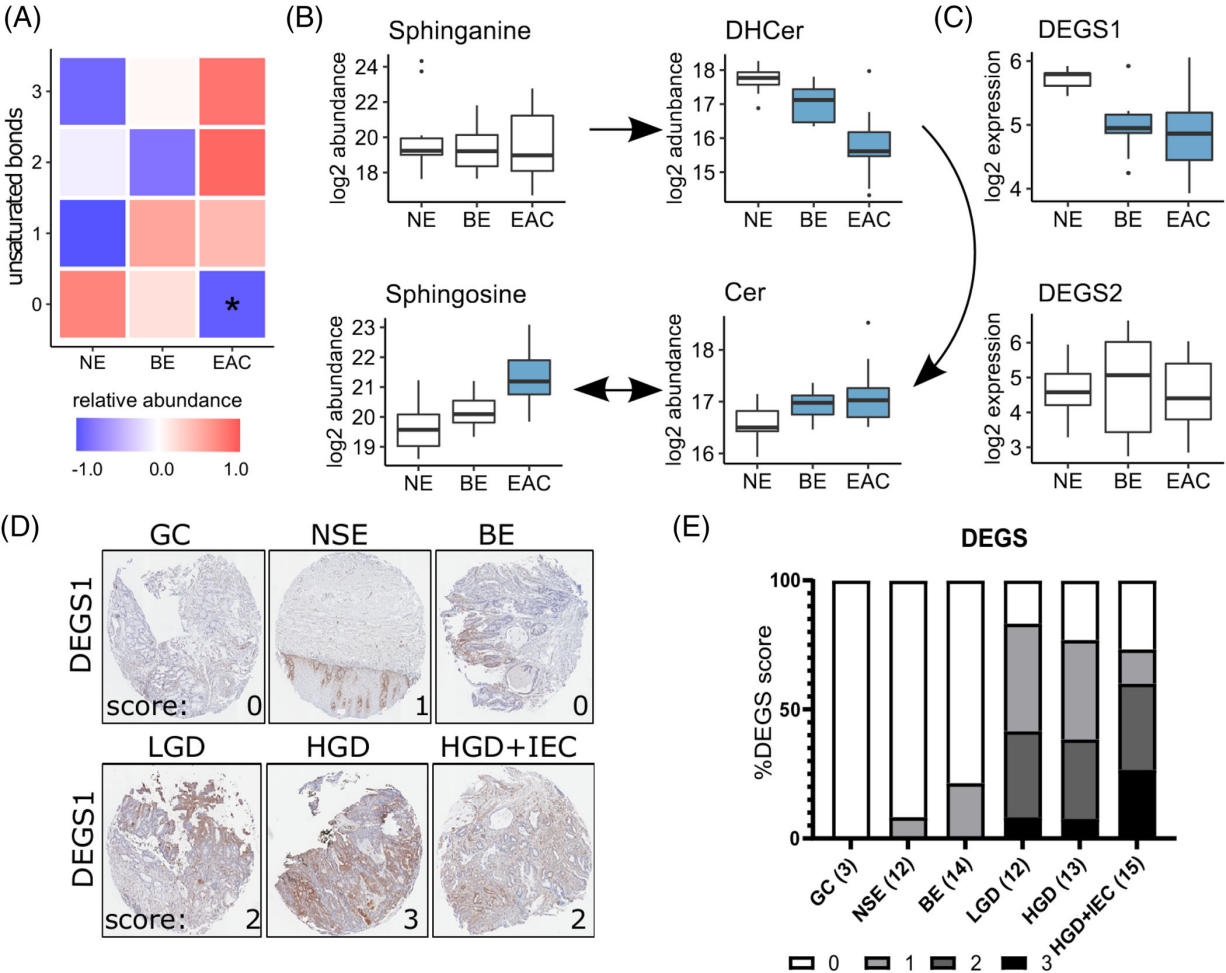
**Ceramide desaturation is altered in esophageal adenocarcinoma (EAC) progression**. (A) Lipidomics data highlight a progressive increase in unsaturated ceramide species in EAC. (B) Changes in sphinganine, DHCer, Cer, and sphingosine appear to be unrelated to changes in DEGS1 and DEGS2 transcript levels (C) across normal epithelium (NE), Barrett's esophagus (BE), and esophageal adenocarcinoma (EAC) tissues. Data are represented using boxplots, with Mann‐Whitney U test or one‐way ANOVA with Benjamini‐Hochberg adjustment, with significance (*p* < .05) from NE represented by shading in the boxplots. Lipid data: NE *n* = 10, BE *n* = 8, EAC *n* = 9. Transcript data: NE = 7, BE = 9, EAC n = 8. (D) Biopsies were stained and scored for DEGS1 protein on a 4‐point scale (0‐3): with representative areas to highlight normal squamous epithelium (NSE), non‐dysplastic Barrett's esophagus (BE), low‐grade dysplasia (LGD), high‐grade dysplasia (HGD), HGD with intraepithelial carcinoma (HGD + IEC) in 1.5 mm cores indicated with black shapes. Scoring of the immunohistochemistry data is shown in (E) (n in brackets). Ordinal logistic fit provided significant changes between BE and LGD (*p* = .002)

**FIGURE 7:**
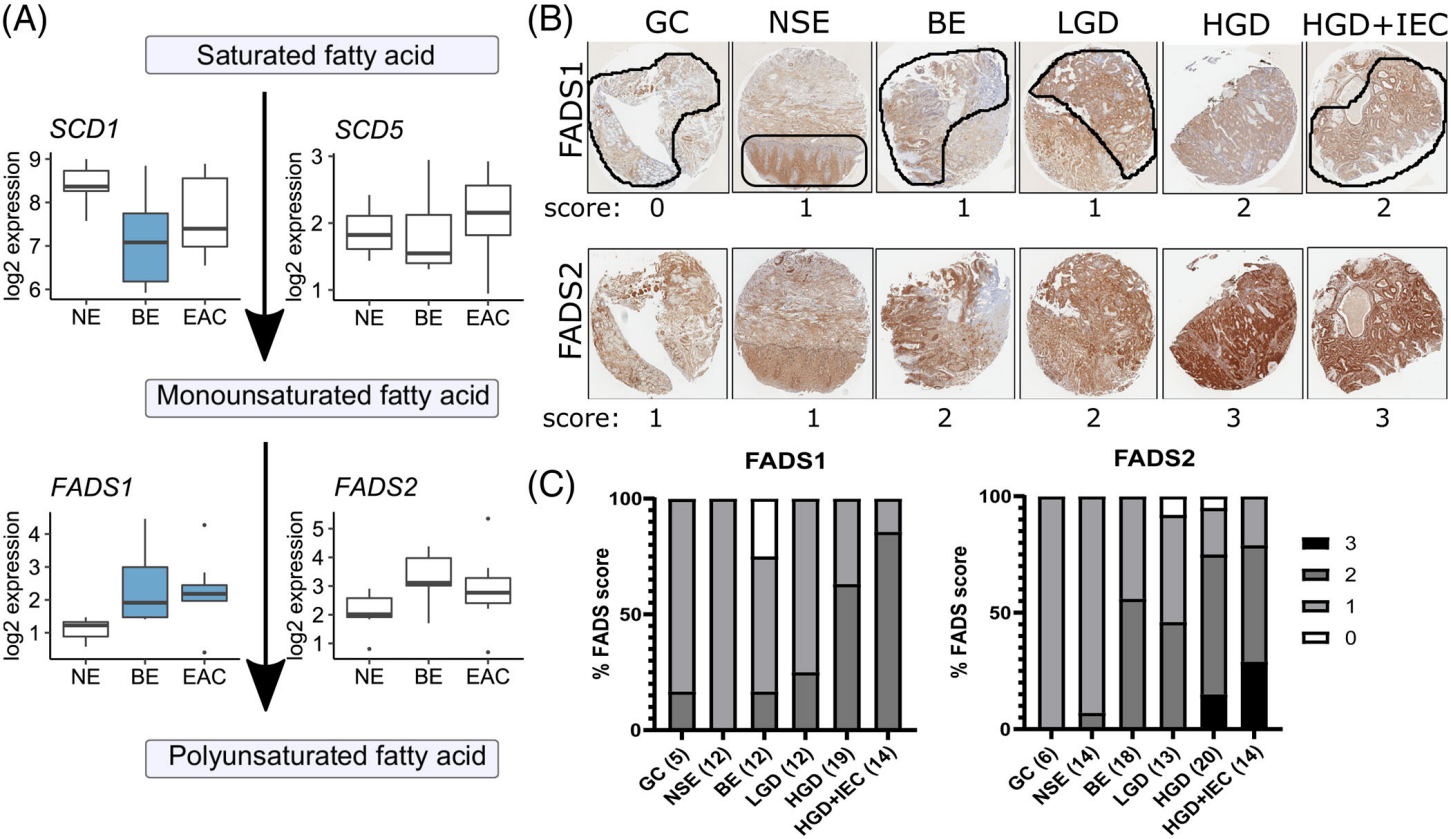
**Lipid unsaturation changes with esophageal adenocarcinoma (EAC) progression, linked to desaturase proteins**. (A) RNA expression of enzymes involved in lipid desaturation, taken from an independent cohort of patient biopsies representing normal esophagus (NE, *n* = 7), Barrett's esophagus (BE, *n* = 9), and esophageal adenocarcinoma (EAC, *n* = 8). Data are represented using boxplots, with Mann‐Whitney U test or one‐way ANOVA with Benjamini‐Hochberg adjustment, with significance (*p* < .05) from NE represented by shading in the boxplots. (B) Scoring of immunohistochemistry for FADS1 and FADS2 was performed on a 4‐point scale (0–3) with representative areas (indicated with black shapes) to highlight normal squamous epithelium (NSE), non‐dysplastic Barrett's esophagus (BE), low‐grade dysplasia (LGD), high‐grade dysplasia (HGD), HGD with intraepithelial carcinoma (HGD + IEC) in 1.5 mm cores Pooled scores (n shown in brackets) are shown in (C). Effect likelihood ratio tests provided p values of < .0001 for both FADS1 and FADS2. Ordinal logistic fit provided no significant sequential changes during disease progression for FADS1, and significant changes between NSE and BE (*p* = .024), and LGD and HGD for FADS2 (*p* = .042)

From the integrated pathway analysis, and based on the availability of validated commercial antibodies, we next used IHC to validate selected enzymes in a larger cohort representing additional stages of EAC pathogenesis. We generated a tissue microarray of esophageal biopsies from BE patients comprising normal squamous epithelium (NSE), gastric cardia (GC), BE, low‐grade dysplasia (LGD), high‐grade dysplasia (HGD), and HGD with intraepithelial carcinoma (HGD+IEC) phenotypes, as evaluated by a specialist pathologist. GC was added as an additional control as it was recently identified as the source of BE.^27^ Figures 6 and 7 compare and integrate the transcriptome and IHC findings for DEGS and FADS (FADS1 and FADS2), respectively. The transcription factor Nrf2, which is the master regulator of antioxidant and detoxifying defense genes activated by both ROS^28^, ^29^, ^30^ and bile acids^29^ was detected in the esophageal epithelium but no significant change was detected in the intensity or localization of Nrf2 during the progression of EAC disease (Figure S6B).

### 2.5 Integrated analysis indicates changes in sphingolipid unsaturation linked to increased dihydroceramide desaturase

The lipidomics data showed an increase in unsaturation of sphingolipids in BE and EAC, with a significant loss of saturated species (zero double bonds, Figure 6A). This specifically reflects decreased ceramides with a saturated backbone (DHCer), with a concomitant increase in ceramide species with a monounsaturated backbone (Cer, Figure 6B). While this pattern suggests an increased activity of the desaturase, we observed a significant decrease in the transcript levels of DEGS1, and no changes in DEGS2 (Figure 6C). Interestingly, IHC staining of DEGS1 protein (Figure 6D) revealed an increase along the stages of EAC pathogenesis (Figure 6D,E), with effect likelihood ratio tests providing *p*‐values of < .0001. Ordinal logistic fit indicated significant changes between NSE and BE (*p* = .0019) for DEGS.

### 2.6 Integrated analysis shows changes in polyunsaturated fatty acids linked to increased FADS isoforms

With respect to fatty acid desaturation enzymes, our transcriptome data detected SCD1, SCD5, FADS1, and FADS2. As depicted in Figure 7A, saturated fatty acid desaturation is carried out by the Δ9‐desaturase SCD (Stearoyl‐CoA Desaturase), which introduces a single double bond between C9 and C10 of saturated long‐chain acyl‐CoAs. Polyunsaturated fatty acids from the diet can be further desaturated by Δ5‐desaturase, encoded by the FADS1 gene, or Δ6‐desaturase (FADS2) (Figure 7A); these cannot be generated *de novo* in mammals. Recent data suggest that FADS can also use saturated fatty acids as a substrate.^31^ The transcriptome data showed an increase in FADS in the sequence NE‐BE‐EAC, with the change in FADS1 statistically significant, and a decrease in SCD1 which is only statistically significant in BE (Figure 7A). The lipidomics data showed a progressive upregulation of lipids with four or more double bonds, and a progressive decrease in lipids with 0–3 double bonds, in BE and EAC (Figure 4C and Figure S3), which is consistent with the pattern of FADS transcripts. In agreement with the transcriptome data, the IHC data showed increased scoring for FADS1 and FADS2 through the progression from NSE and GC to HGD+IEC (Figure 7B), with examples of FADS2 staining shown in Figure 7C. Effect likelihood ratio tests provided *p*‐values of <.0001 for both FADS1 and FADS2. Ordinal logistic fit analysis was conducted, which revealed significant changes between NSE and BE (*p* = .024), and LGD and HGD (*p* = .042) for FADS2, but no significant changes for FADS1.

### 2.7 Functional assessment of FADS2 in EAC

**FIGURE 8:**
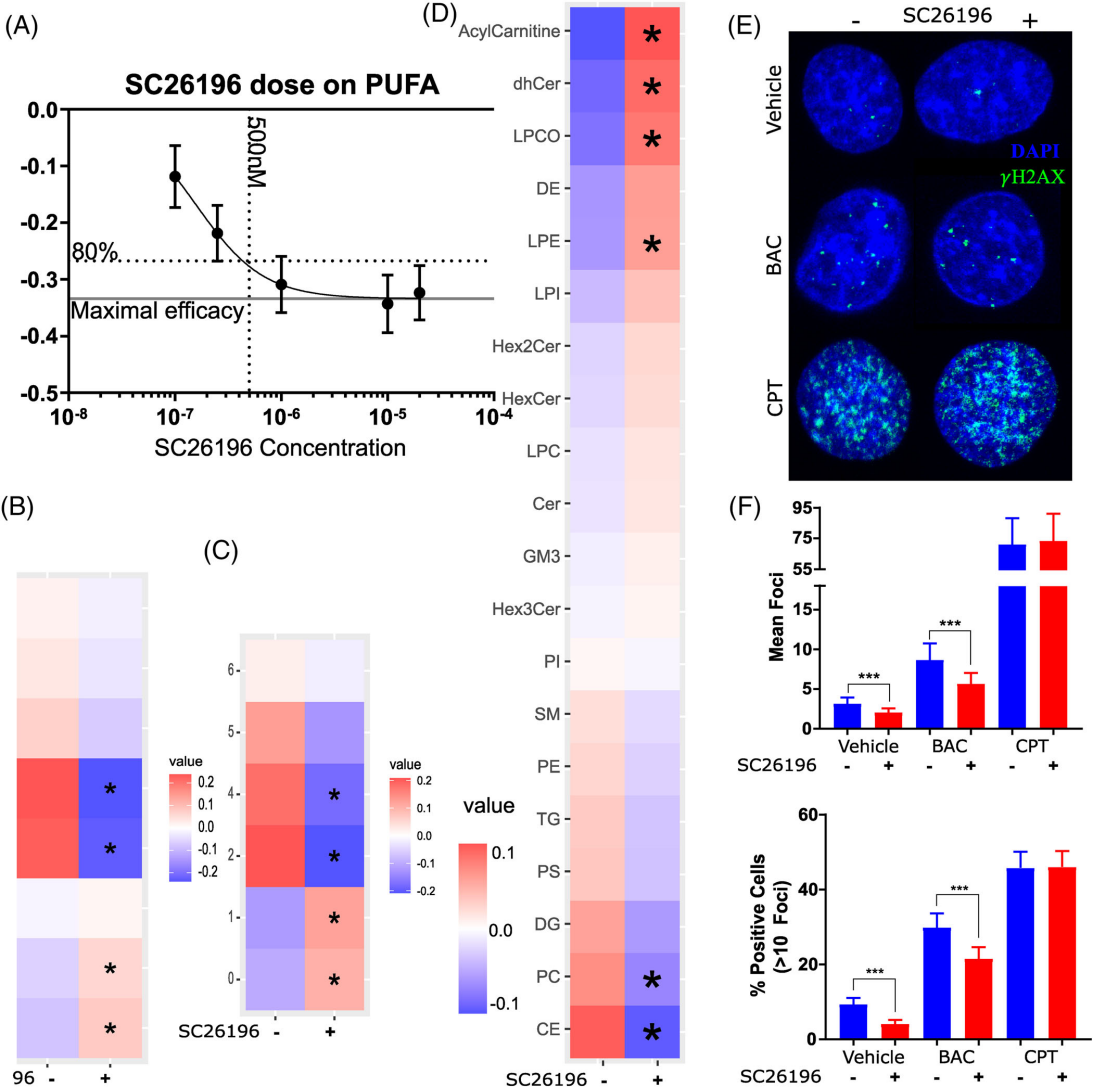
**Fatty acid desaturases (FADS) inhibition alters lipid profile and DNA damage in FLO‐1 EAC cells**. FLO‐1 EAC cell line was treated with FADS2 inhibitor SC26196 for 48 hours before treatment with a bile acid cocktail (BAC, 1000 μM final, including an equimolar mixture of sodium salts of taurocholic acid, deoxycholic acid, glycodeoxycholic acid, glycocholic acid, and glycochenodeoxycholic acid) at pH 4 for 20 min, camptothecin (CPT, 1 μM, pH 7) for 60 min, or left untreated. (A) Dose titration of SC26196 for reduction of lipids with three or four double bonds. 500 nM SC26196 provides 80% of maximal response. (B) 500 nM SC26196 decreases lipids with three or four double bonds in lipids with two chains compared to vehicle in FLO‐1 cells, and (C) in lipids with two or four double bonds in single‐chain lipids. (D) Changes in lipid class were also induced by 48 h exposure to 500 nM SC26196. *, *p* < .05 change from control. (E) Immunofluorescence staining of γH2AX foci (green) to visualize dsDNA damage, with the nuclei stained blue (DAPI). (F) The mean foci per cell was increased with BAC and 1 μM CPT, but SC26196 treatment decreases the mean foci per cell in BAC and vehicle. (G) Similarly, the % of cells showing more than five foci per cell was increased by BAC and CPT, while FADS inhibition by SC26196 decreased the number of positive cells in vehicle and BAC. Data from biological quadruplicates and technical duplicates. ***, *p* = .001

With the observed increase in FADS and polyunsaturated lipids in EAC development, and previous reports that bile acids induce oxidative DNA damage in the esophagus,^32^, ^33^ we hypothesized that a reduction of polyunsaturated lipids through FADS2 inhibition could reduce sensitivity to bile acids‐induced genotoxicity. To test this hypothesis, we treated the FLO‐1 EAC cell line with FADS2 inhibitor SC26196, with or without a bile acid cocktail (BAC) to mimick reflux. An initial SC26196 dose titration experiment was conducted to identify the dosing, which found half‐maximal inhibitory concentration (IC50) for reduction of lipids with 3 and 4 double bonds to be 156 nM SC26196 (Figure 8A). The dose of 500 nM was selected to achieve 80% of the maximal response. The effect and specificity of 500 nM SC26196 on FLO‐1 cell lipidome are shown in Figure 8B,C. As expected, SC26196 treatment induced a decrease in lipids with three and four double bonds, and a small increase in saturated and monounsaturated lipids (Figure 8B). In addition, SC26196 also induced a significant decrease in Cer and PC, and an increase in DHCer, acylcarnitine, LPCO, and LPE (Figure 8D). DNA damage was measured by immunofluorescence microscopy for γH2AX with a number of foci indicating double‐strand DNA (dsDNA) breaks (Figure 8E). The chemotherapy agent camptothecin (CPT) served as assay positive control. As expected, based on previous reports,^32^, ^33^ treatment with BAC at pH4 increased both the average number of foci per cell (Figure 8F), as well as the number of cells with greater than 5 foci (Figure 8G). Blocking FADS2 with SC26196 decreased γH2AX foci in both baseline and BAC treated cells (Figure 8F,G). On the other hand, SC26196 had no effect on γH2AX foci induced by CPT (Figure 8F,G). These results demonstrate a link between polyunsaturated lipids/FADS2 activity and protection from bile acid‐induced DNA damage in FLO‐1 cells.

## 3 DISCUSSION

Through lipid‐focused integrated tissue multiomic analysis, we discovered progressive changes in polyunsaturated lipids, lipid chain lengths, and specific lipid classes during disease pathogenesis. Candidate genes/proteins from the relatively small cohorts for lipidomic, proteomic, and transcriptomic analyses were verified through immunohistochemical staining of a larger cohort to confirm the increased expression of FADS2 and DEGS1 enzymes in EAC patients. A functional link between FADS2 activity, polyunsaturated lipids, and the promotion of bile acid‐induced DNA damage was revealed in vitro studies. Lipid desaturation, therefore, appears to be a key alteration in EAC progression; although proteomic or transcriptomic analysis alone did not detect this. For example, DEGS1 transcript was reduced in EAC, while lipidomic and IHC data indicate an increase in DEGS activity and protein, respectively. Therefore, this study highlights the importance of verifying that transcript alterations translate to the protein level and the utility of multiomics in unveiling pathobiology pathways.

There are several implications of increased lipid desaturation in lipid biosynthesis. The increased availability of lipids can change cellular energetics as a fuel source, a conclusion supported by our proteomic results implicating changes in the TCA cycle and ketogenesis. The changes in the TCA cycle may not be primarily driven by lipid: the Warburg effect has shown that cancer cells take up much more glucose than healthy cells, which would utilize the TCA cycle for energy,^34^ thereby generating ROS and activating anti‐oxidant pathways including Nrf2.^35^ The double bonds in polyunsaturated lipids are susceptible to attack by ROS, which is increased in most cancers.^36^ In EAC, bile acids in combination with low pH consistent with GERD induce genotoxic oxidative stress.^7^, ^32^, ^33^ Here, we report FADS2 inhibition protects against bile acid‐induced DNA damage, concomitant with a reduction in polyunsaturated lipids with 4 or more double bonds, and a change in some lipid classes. In addition to DNA damage and energetics, polyunsaturated lipids can have many other effects related to signaling,^4^ membrane fluidity,^37^ and inflammation.^38^ It is tempting to speculate that lipid metabolism re‐wiring simultaneously activates the cancer hallmarks of deregulating cellular energetics, sustaining proliferative signaling, evading growth suppressors, immune responses, resisting cell death, and genome instability and mutation.^39^

Genotoxic aldehydes^10^ and exhaled aldehydes are increased in EAC patients,^10^ likely as a result of peroxidation of the elevated polyunsaturated fatty acids and reduced aldehyde detoxification. ALDHs, including ALDH1A1, ALDH1B1, ALDH2, and ALDH6A1, as well as glutathione S‐transferases, convert genotoxic aldehydes to acetyl‐CoA^40^: this can feed into the TCA cycle^41^ or the ketogenic pathway,^42^ both of which are upregulated in our study. ALDHs can therefore mitigate oxidative stress and may protect cancer cells from oxidative cell death and genotoxicity, as well as contribute to energy production.^43^ Effects of ALDHs on cancer are varied: ALDH1 levels have been linked to chemotherapy response and a higher proliferation rate,^44^ and tumor regression and patient survival in models of esophageal squamous cell carcinoma.^45^ A loss in ALDH3A2 was linked to poor prognosis in EAC,^10^ yet induced ferroptotic cell death in leukemic cells.^46^ Targeting ALDH3A1 in gastric cancers has anti‐cancer effects, and prevented 40% of NADH generation, leading to the conclusion that a large source of β‐oxidation of fatty acids is dependent on ALDH3A1,^47^ and placing ALDH at a unique position of reducing genotoxic effects, altering survival, and providing a fuel source through β‐oxidation in EAC cells. A meta‐analysis highlighted a number of ALDHs that were underexpressed in EAC, and confirmed with qPCR^10^: this contrasts with our proteomics results, which show a decrease in some ALDH with BE, but an increase in many others with EAC. It remains to be determined whether the changes observed in our study reflect an improved ability to deal with genotoxic aldehydes. While ALDH proteins deal specifically with genotoxic aldehydes, our data also highlights changes in other anti‐oxidant pathways, including PRDX, GPX, and catalase, and many of these fall under transcriptional control of Nrf2. Nrf2 protects esophageal cells against ROS^29^ and cell death due to oxidative reflux conditions in BE,^28^ however our data did not show an appreciable change in Nrf2 protein levels or localization in disease progression. This is consistent with previous studies that have demonstrated changes in Nrf2 transcriptional activity without alterations in Nrf2 protein levels or localization.^48^, ^49^ The specific contribution of the antioxidant pathways in this study to genotoxicity, proliferation, and ferroptosis remains to be clarified. However, we show a link between FADS2 inhibition and reduced dsDNA damage, which highlights the role of polyunsaturated lipids in genotoxicity in EAC.

There are several limitations to the current study. Firstly, our global lipidomics method only provides the total number of double bonds without positional information, hence additional research will need to be performed to distinguish between omega‐3 and omega‐6 fatty acids. In addition, our cohorts lacked comprehensive clinical and dietary information. Polyunsaturated fatty acids cannot be generated in vivo, hence dietary input will need to be considered in future studies. We have focused on polyunsaturation in oxidative DNA damage response, but many pathways discovered through the multiomics approach deserve further study: arachidonic acid metabolism^50^ and the TCA cycle are altered in EAC development, and SPMs are involved in the resolution of inflammation in cancer,^51^ and are being investigated as anti‐cancer molecules.^52^ The role of ceramide and DEGS1 requires further investigation to understand its effects in cancer cells, particularly EAC,^53^ and ceramide has already been investigated as a target for cancer therapy.^54^ As SC26196 altered Cer and DHCer, as well as changes in lysophospholipids and acylcarnitine, further characterization is required to determine whether this is a direct effect of FADS inhibition or a secondary effect of the changing lipidome. Lipid peroxidation also deserves further study due to changes in lipid polyunsaturation and anti‐oxidant pathways. Finally, the functional role of polyunsaturated lipids in bile acid‐induced genotoxicity was investigated in a single EAC cell line and should be further examined in other relevant cells and in organotypic cell culture models. Strikingly, our preliminary proteomic assessment of a range of cell types representing BE, HGD, and EAC in 2D culture (Figure S7) highlighted differences in protein expression of antioxidation pathways and unexpected expression of esophageal defenses^55^ such as mucins,^56^, ^57^ cadherins and desmosomes^58^ which were not consistent with human pathology. This may be due to a number of differences between cell lines and biopsies, such as the lack of supporting stroma and environmental stresses, including GERD. GERD was mimicked in cell culture by exposure to bile acids at low pH in our model and others,^59^ to induce oxidative DNA damage as a hallmark of cancer. It would be interesting to investigate the baseline antioxidant protein expression of BE/EAC cell lines with their sensitivity to bile acid‐induced oxidative DNA damage.

While we were undertaking validation studies, an EAC tissue lipidomics study on a cohort from the United Kingdom was published.^11^ While both studies revealed changes in lipid classes, chain length, and unsaturation during EAC development, in contrast to Abbassi‐Ghadi et al.,^11^ our data did not show significant change for PG, and the PG biosynthetic genes LPGAT and PGS1. Furthermore, the lipid desaturase enzyme validated in the two studies also differs: SCD in Abbassi‐Ghadi et al.,^11^ FADS2 in this study. These differences may be due to genetic or environmental factors between the two cohorts and remains to be evaluated in future studies.

In conclusion, we show results from three independent cohorts, reflecting lipidomic, proteomic, transcriptomic, and immunohistochemical analyses that highlight changes in lipid metabolism and antioxidant responses in the progression from normal esophageal tissue, through BE, to EAC. An increase in polyunsaturated lipids was linked to increased desaturase proteins, detected by IHC, but not always indicated in transcriptome data. Importantly, we also show that blocking desaturation by FADS2 inhibition protects an EAC cell line from bile acid‐induced DNA damage, providing an avenue for novel prevention and treatment strategies in EAC.

## 4 METHODS

### 4.1 Patient cohorts

Human Research Ethics approvals were obtained from the University of Queensland Human Research Ethics Committee (approval number 2008002308) and the QIMR‐Human Research Ethics Committee (approval number P1188).

### 4.2 Patient samples for lipidomic and proteomic analysis

Patient biopsy samples were obtained from the PROBE‐NET (Progression of Barrett's Esophagus – Network) study and from Professor Andrew Barbour's surgical oncology group (SOG), all participants gave written informed consent. Ten esophageal biopsies were selected for BE and EAC, as well as the healthy NE, initially as determined by the endoscopist. Following endoscopic/surgical removal, biopsies were collected in screw cap tubes containing RNAlater and stored at 4°C during the operation, and at –80°C for long‐term storage. The collected samples represented the disease stages BE and EAC, as well as the healthy squamous epithelial controls. BE tissues were based on histology of an adjacent biopsy from the same patient as tissues with a columnar phenotype and intestinal metaplasia were selected, one EAC sample was excluded as histology indicated squamous cell carcinoma instead of EAC.

### 4.3 Patient samples for transcriptomic analysis

The Brisbane RNAseq dataset contains RNA sequencing performed on nine BE, eight EAC, and seven matched NE tissue samples. The samples were collected and stored in RNAlater prior to RNA extraction and sequencing.

### 4.4 Patient biopsies for IHC

Formalin‐fixed, paraffin‐embedded archival esophageal tissue samples were retrieved from 84 patients at Envoi Pathology. In addition, five normal tissues from the non‐malignant portion of the esophagus were resected from surgery and used as controls. No patients received pre‐operative chemotherapy or radiotherapy. All tumor tissues were confirmed as esophageal tissue by hematoxylin and eosin (H&E) staining after surgical resection.

### 4.5 Tissue sample preparation for lipidomic and proteomic analysis

Polar metabolites (lipids) and proteins were extracted from the same piece of biopsy tissue by homogenization in ice‐cold methanol with a steel bead (TissueLyzer LT, Qiagen, Melbourne Australia), then washed and dried under nitrogen, resuspended in methanol and 50 μg/ml BHT. Tissues were homogenized by three cycles of vortex mixing, freezing in liquid nitrogen, and sonication (Grant XUB18 bath sonicator, Grant Instruments, Cambridgeshire, UK)^15^ and subjected to global lipidomics and proteomics analyses. Details are provided in Supporting Information.

**Lipidomics**: Untargeted (positive ionization mode) and targeted lipidomics were performed according to previously published methods.^15^, ^68^ Additionally, untargeted lipidomics in negative ionization mode was performed using the same instrument and parameters. Untargeted lipidomics data processing was performed using XCMS^60^ and lipid identification was performed using MS‐DIAL version 2.64^61^ and LipidMatch.^62^ All data were processed using probabilistic quotient normalization^63^ and log2 transformation, followed by missing value imputation using a deterministic minimum value approach. Lipid class representation during the BE/EAC disease progression was evaluated using hypergeometric distribution analysis. Overrepresentation p‐values were adjusted using Benjamini‐Hochberg correction.^64^ The mass spectrometry patient lipidomics data have been deposited to the MetabolomicsWorkbench repository ^65^ with the study identifier ST001942.

**Proteomics**: Proteins were collected by centrifugation of the methanol extract and resuspended in lysis buffer containing 1% sodium deoxycholate. The protein concentration was determined and the 20 μg sample was digested with trypsin. The resulting peptides were collected for analysis on a Thermo Easy‐nLC 1000‐coupled Orbitrap QE plus mass spectrometer. Spectrum data were analyzed with MaxQuant version 1.6.1.0 with the human Swiss‐Prot database containing 20 258 reviewed proteins (downloaded February 2018).The mass spectrometry proteomics data have been deposited to the ProteomeXchange Consortium via the PRIDE partner repository^66^ with the dataset identifier PXD028380.

### 4.6 Immunohistochemical staining and scoring

Immunohistochemical staining was performed on formalin‐fixed paraffin‐embedded tissues in a tissue microarray format, using antibodies against DEGS1 (Abcam#ab167169), FADS1 (Abcam#ab236672), FADS2 (Abcam#ab232898) and Nrf2 (Abcam#ab137550). Staining details are provided in Supporting Information. Staining intensities in GC, NSE, BE, LGD, HGD, and intraepithelial carcinoma (IEC) were scored by a specialist gastrointestinal pathologist. The phenotype was determined on the basis of H&E‐stained sections, according to the standard diagnostic procedure. Phenotypes occasionally changed between sequential sections, such that slightly different numbers exist for each analysis. Scoring was based on the phenotype in the region of interest: for example, NSE refers to only the normal squamous epithelium in the section: this is in contrast to the omics data which use the whole biopsy, where normal esophageal tissue may include stroma, submucosal glands, as well as epithelium. Each component, if present in the tissue, was scored separately using a 4‐grade assessment of intensity (0 no staining, 1+ weak staining, 2+ moderate staining, 3+ strong staining). Where the staining was non‐uniform in a component, the maximum intensity of staining was used for the score, providing at least 10% of the cells of that component stained to this intensity.

### 4.7 Tissue RNA sequencing

RNAs were extracted using previously published methods by Ross‐Innes et al.^67^ and were analyzed using the Illumina HiSeq 2500 high‐throughput sequencing system (Illumina Inc., San Diego, USA). STAR aligner (version 2.5.2a) was used to align the paired‐end reads to the human reference genome version GRCh37. Cutadapt (version 1.9) was used to trim the sequence adaptors and gene expression was estimated using RSEM (version 1.2.30). Trimmed mean of *M*‐values normalization was applied to the expression data using the R package edgeR.

### 4.8 Cell culture experiments

Cell culture details are provided in Supporting Information. FLO‐1 cells were used to determine dsDNA damage in response to bile acids, pH 4, and FADS inhibition. To mimic the composition of GERD bile acids in the distal esophagus, a bile salts cocktail containing an equimolar mixture of sodium salts of taurocholic acid, deoxycholic acid, glycodeoxycholic acid, glycocholic acid, and glycochenodeoxycholic acid was prepared as previously described.^70^ All experiments utilized a final bile salt cocktail concentration of 1000 μM diluted in pH 4 media. The FADS2 inhibitor SC26196 (Sigma Aldrich) was dissolved in DMSO, which was also used as the vehicle‐only negative control.

For targeted lipidomics, cells were harvested, counted, and washed twice in cold PBS. Cell pellets (1 × 10^6^ cells) were stored at ‐80°C until lipid extraction, and targeted lipidomics were performed using a method modified from Huynh et al.^68^ on an Agilent Technologies 1290 Infinity II LC System with a Zorbax Eclipse Plus C18 RRHD 2.1 × 100 mm 1.8μm column, coupled to an Agilent 6470A Triple Quadrupole Mass Spectrometer via Jet Stream ionization source. Additional information is included in Supporting Information. The mass spectrometry cell lipidomics data have been published on Panoramaweb^71^ repository at https://panoramaweb.org/SC26196FADSinhibitionFLO‐1.url

For assessment of DNA damage, γH2AX foci were detected by indirect immunofluorescence as previously described^69^ with modifications detailed in Supporting Information. Image analysis, including automatic cell detection and γH2AX foci quantification, was performed using open‐source software, QuPath 0.2.3. All statistical analyses adopted a mixed effect model, considering variation between biological replicates as random effect whilst each condition and the presence of SC26196 were considered fixed effect variables. When considering the mean foci count per cell, the data were fitted with a negative binomial regression to account for the negative over‐dispersion. Cells were considered to show DNA damage when there were more than 5 foci in a cell, and data were fitted with a logistic regression model.

## FUNDING INFORMATION

Translational Research Institute Spore Grant, University of Queensland International Postgraduate Research Scholarship. PROBE‐NET was funded by the National Health and Medical Research Council (NHMRC) of Australia (NHMRC APP1040947) and Cancer Council NSW (SRP 08‐04).

## CONFLICT OF INTEREST

The authors declare no conflict of interest.

## Supporting information



Supporting Information

Supporting Information

Supporting Information
