# Supplementary material for: Elevation of fatty acid desaturase 2 in esophageal adenocarcinoma increases polyunsaturated lipids and may exacerbate bile acid‐induced DNA damage
Source: Clin Transl Med. 2022 May 12;12(5):e810. doi: 10.1002/ctm2.810 (PMC9099135; doi:10.1002/ctm2.810)
Supplement: Supplementary file 1 — Supporting Information [file CTM2-12-e810-s002.pdf]

Supplementary information for

Elevation of fatty acid desaturase 2 in esophageal adenocarcinoma increases polyunsaturated lipids and may exacerbate bile acid-induced DNA damage

Jeffrey Molendijk[1,2#], Cathryn M. Kolka[2#], Henry Cairns[2], Sandra Brosda[1], Ahmed Mohamed[1,2], Alok K. Shah[1,2], Ian Brown[3], Mark P. Hodson[4], Thomas Hennessy[1,5], Guanghao Liu[2], Thomas Stoll[2], Renee S. Richards[2], Michael Gartside[1], Kalpana Patel[1], Nicholas J. Clemons[6,7], Wayne A. Phillips[6,7], Andrew Barbour[1], Johan A. Westerhuis[8], Michelle M. Hill[1,2*]

CONTENTS:

Table S1. List of lipid abbreviations P2

Table S2. Lipidomics data see separate excel sheet

Table S3. Patient cohort information for proteomics/lipidomics and transcriptomics cohorts. P3

Table S4. Joint loadings from JIVE analyses see separate excel sheet

Figure S1. Sample exclusion process. P4

Figure S2. JIVE decomposition component heatmaps. P5

Figure S3: Unsaturation enrichment and chain length analysis for lipids with 1 or 3 fatty acids. P6

Figure S4. Patient tissue proteomics data for selected redox proteins. P7

Figure S5. Patient tissue transcriptomic data for selected redox genes. P8

Figure S6. NRF2 is not altered in EAC progression. P9

Figure S7. Comparison of human tissue and cell line proteome for BE and EAC. P10

Supplementary Methods

Lipidomics: patient samples

Lipidomics: cell samples

Proteomics

Immunohistochemical staining

Cell culture

Immunofluorescence

Table S1. List of abbreviations for lipids assessed in lipidomics analysis.

**Abbreviations for lipids**	
CE	Cholesteryl ester
Cer	Ceramide
Cer-AS	Ceramide alpha-hydroxy fatty acid-sphingosine
Cer-BS	Ceramide beta-hydroxy fatty acid-sphingosine
DG	Diacylglycerol
DGDG	Digalactosyldiacylglycerol
DHCer	Dihydroceramide
ether-LPC	Ether-linked lysophosphatidylcholine
ether-PC	Ether-linked phosphatidylcholine
ether-PE	Ether-linked phosphatidylethanolamine
FAHFA	Fatty acid ester of hydroxyl fatty acid
HBMP	Hemibismonoacylglycerophosphate
HexCer-AP	Hexosylceramide alpha-hydroxy fatty acid-phytosphingosine
HexCer-NS	Hexosylceramide non-hydroxy fatty acid-sphingosine
LPA	Lysophosphatidic acid
LPC	Lysophosphatidylcholine
LPCO	Ether-linked lysophosphatidylcholine
LPE	Lysophosphatidylethanolamine
LPI	Lysophosphatidylinositol
LPS	Lysophosphatidylserine
MGDG	Monogalactosyldiacylglycerol
OxLPC	Oxidized lysophosphatidylcholine
PA	Phosphatidic acid
PC	Phosphatidylcholine
PE	Phosphatidylethanolamine
PEtOH	Phosphatidylethanol
PG	Phosphatidylglycerol
PI	Phosphatidylinositol
PS	Phosphatidylserine
SM	Sphingomyelin
So	Sphingosine
TG	Triacylglycerol

Table S2. Lipidomics data **see separate excel sheet**

Table S3. Patient cohort information for proteomics/lipidomics and transcriptomics cohorts.

	**Proteomics/Lipidomics**				**Transcriptomics**		
**Variable**	**NE**	**BE**	**EAC**		**NE**	**BE**	**EAC**
**Samples #**	10	8	9		7	9	8
	**Age**				**Age**		
**Mean (SD)**	64 (13)	62 (14)	65 (10)		64 (15)	64 (16)	64 (12)
**Median (Min/Max)**	70 (41, 78)	65 (41,78)	67 (46, 77)		66 (38,79)	67 (38,79)	65 (42,81)
	**Sex**				**Sex**		
**Male**	9	7	9		5	8	7
**Female**	1	1	0		1	0	1
**Unknown**	**0**	0	0		1	1	0
	**BMI**				**Body weight (kg)**		
**Mean (SD)**	31 (5)	31 (6)	27 (5)		74 (11)	80 (9)	85 (27)
**Median (Min/Max)**	31 (23, 40)	30 (23, 40)	26 (22, 39)		77 (60,84)	83 (68,91)	73 (60, 140)
	**Smoking**				**Smoking**		
**Never**	3	3	3				
**Previous**	7	5	4				
**Current**	0	0	2				
	**Alcohol consumption**				**Alcohol consumption**		
**None**	4	4	1		2	2	2
**< 10g**	1	1	6		1	2	2
**> 10g**	5	3	2		0	2	2
**Unknown**	**0**	0	0		4	3	2

Table S4. Joint loadings from JIVE analyses **see separate excel sheet**

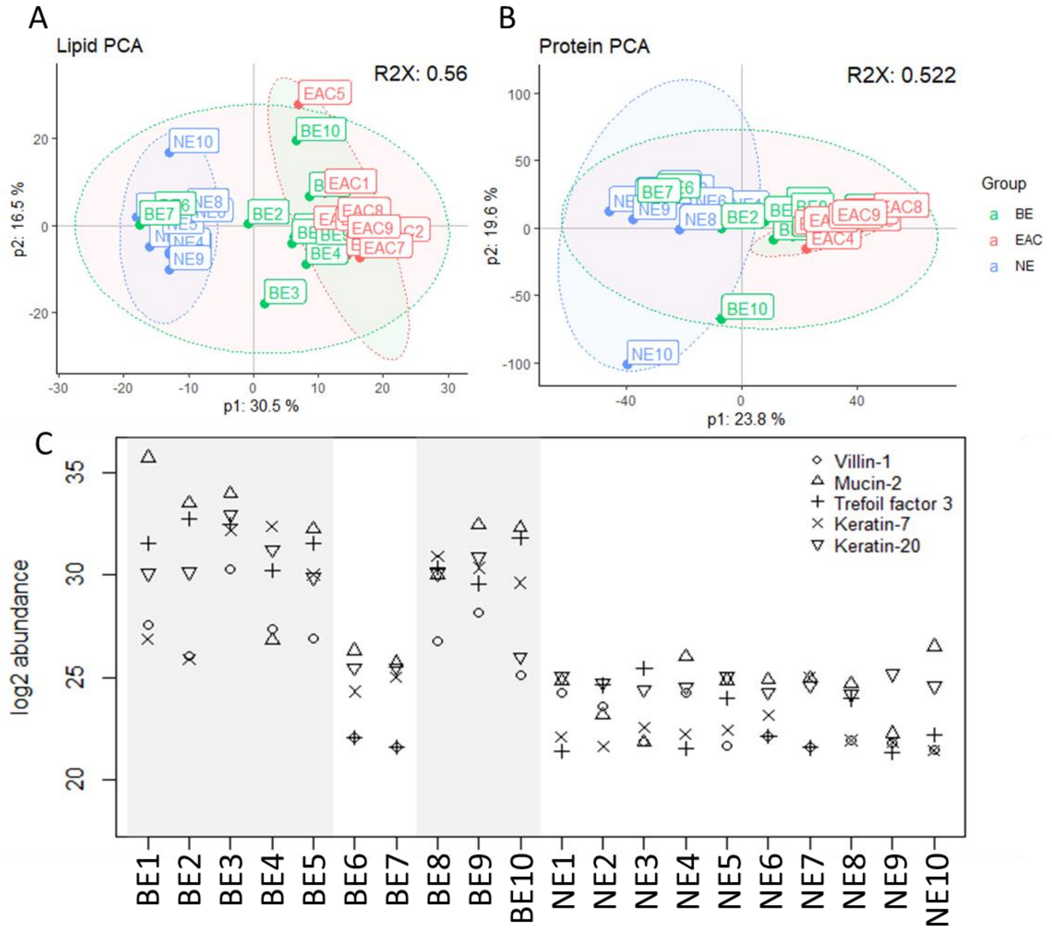

Figure S1. Sample exclusion process. PCA score plots of patient biopsy (A) lipidomics data (n = 586 lipids) and (B) proteomics data (n = 3387 proteins). Dot color indicates the sample disease phenotype. Data was autoscaled prior to model generation. Ellipse: Hotelling's T2 (95%). Log 2 abundance of protein markers for intestinal metaplasia, analysed using MS proteomic techniques on biopsy tissue. Two BE samples (BE6 and BE7) clustered with normal squamous epithelium in both lipid and protein PCA plots. When analysed for protein abundance for intestinal metaplasia markers (C), these samples resembled squamous epithelium, and were excluded from further analyses.

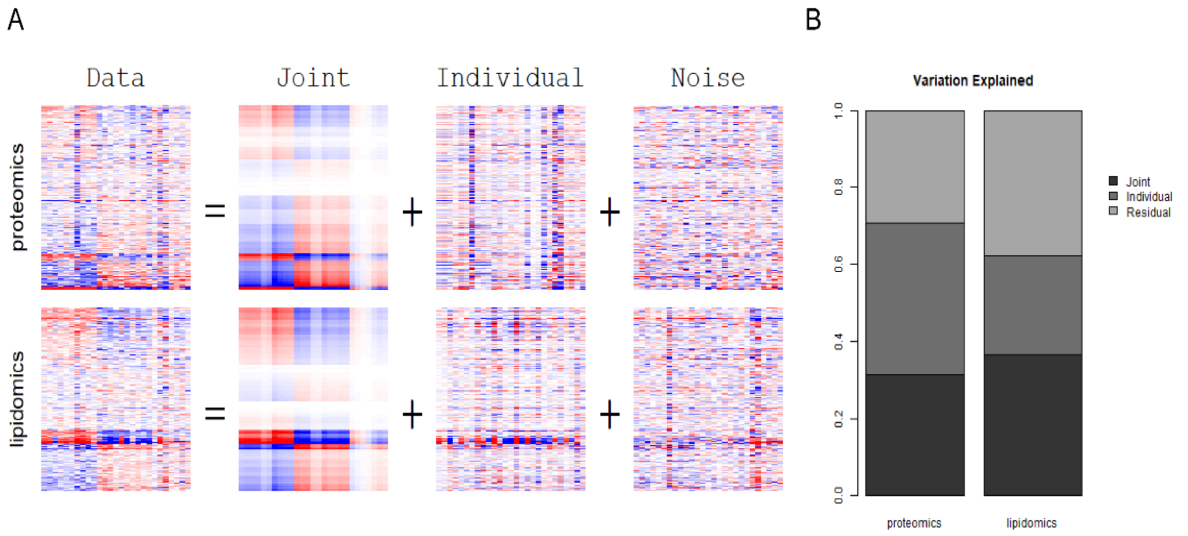

Figure S2. JIVE decomposition component heatmaps. (A) The variation in the proteomics and lipidomics datasets are divided into joint variation, individual variation and residual variation. (B) The joint variation explains 31% and 37% of the total variation in the proteomics and lipidomics datasets respectively.

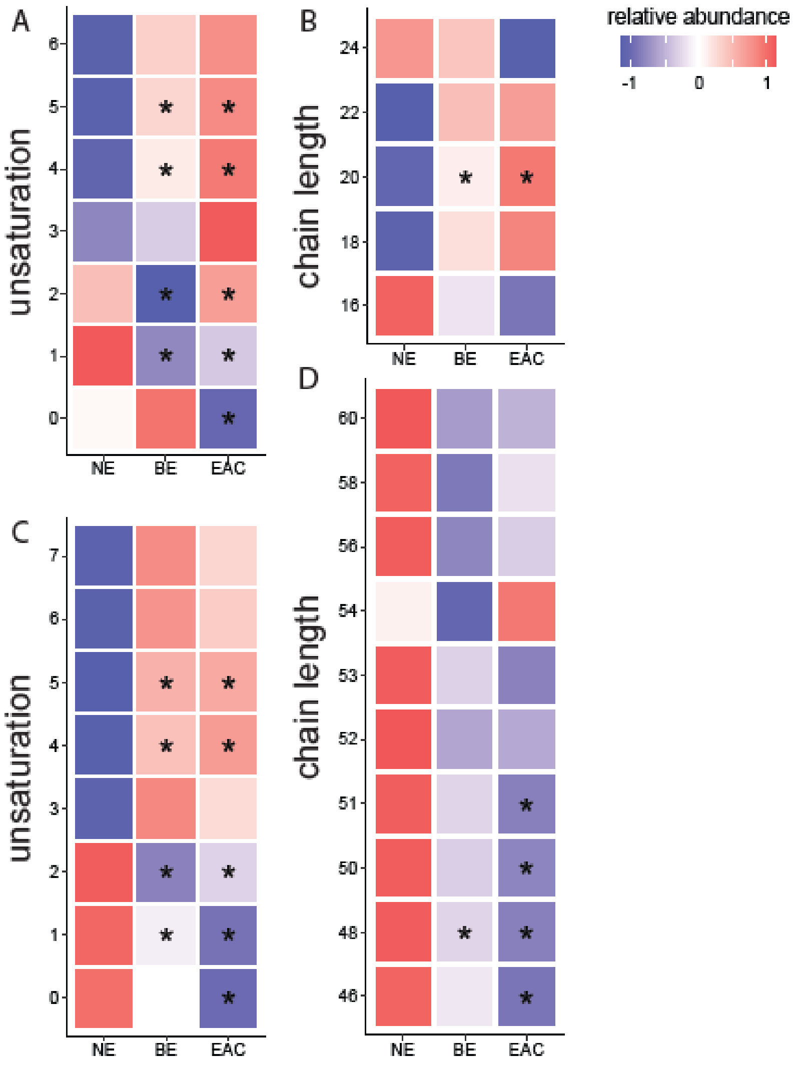

Figure S3: Unsaturation enrichment and chain length analysis for lipids with 1 or 3 fatty acids. (A) Unsaturation enrichment for lipids with 1 fatty acid (including lysophosphatidylethanolamine, lysophosphatidylcholine, lysophosphatidylinositol, single chain fatty acids) demonstrates an increase in lipids with unsaturation of 4 and 5, and a decrease in saturated lipids from normal esophagus (NE, n=10), through Barrett's Esophagus (BE, n=8) to esophageal adenocarcinoma (EAC, n=9), as well as an increase in lipids with a chain length of 20 (B). (C) Unsaturation enrichment for lipids with 3 fatty acid (triglyceride) demonstrates an increase in lipids with unsaturation of 4 and 5, and a decrease in saturated lipids, (D) Chain length analysis for lipids with 3 fatty acids shows a decrease in triglycerides with a shorter total chain length. NES: normalized enrichment score. * p.adj < 0.05.

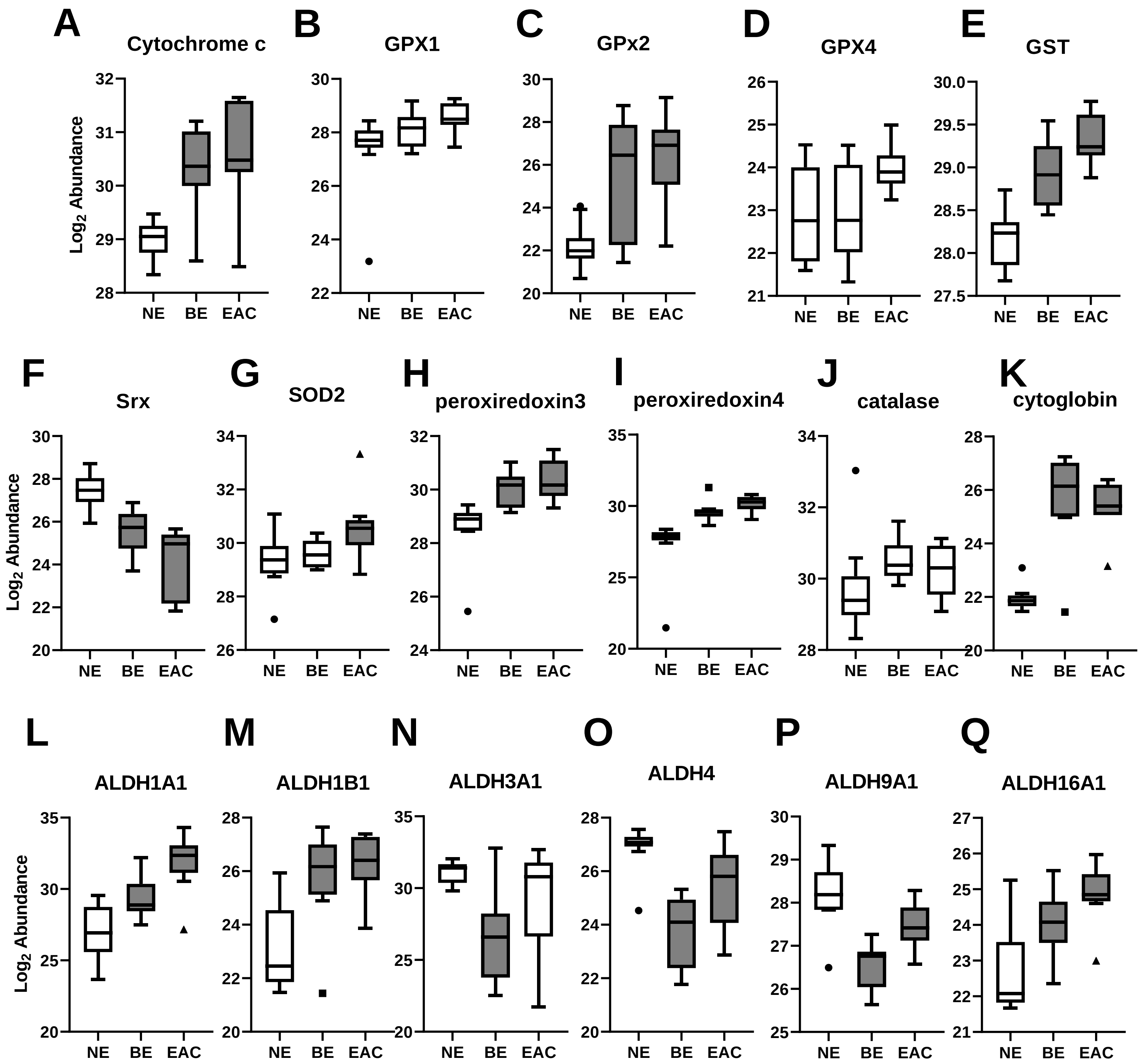

Figure S4. Patient tissue proteomics data for selected redox proteins. Log_2 abundance protein levels for a range of redox enzymes in normal esophagus (NE, n=10), Barrett's Esophagus (BE, n=8) and esophageal adenocarcinoma (EAC, n=9). Shaded boxes indicate significance (P<0.05) by one way ANOVA compared to NE, overrepresentation p-values were adjusted using Benjamini-Hochberg correction.

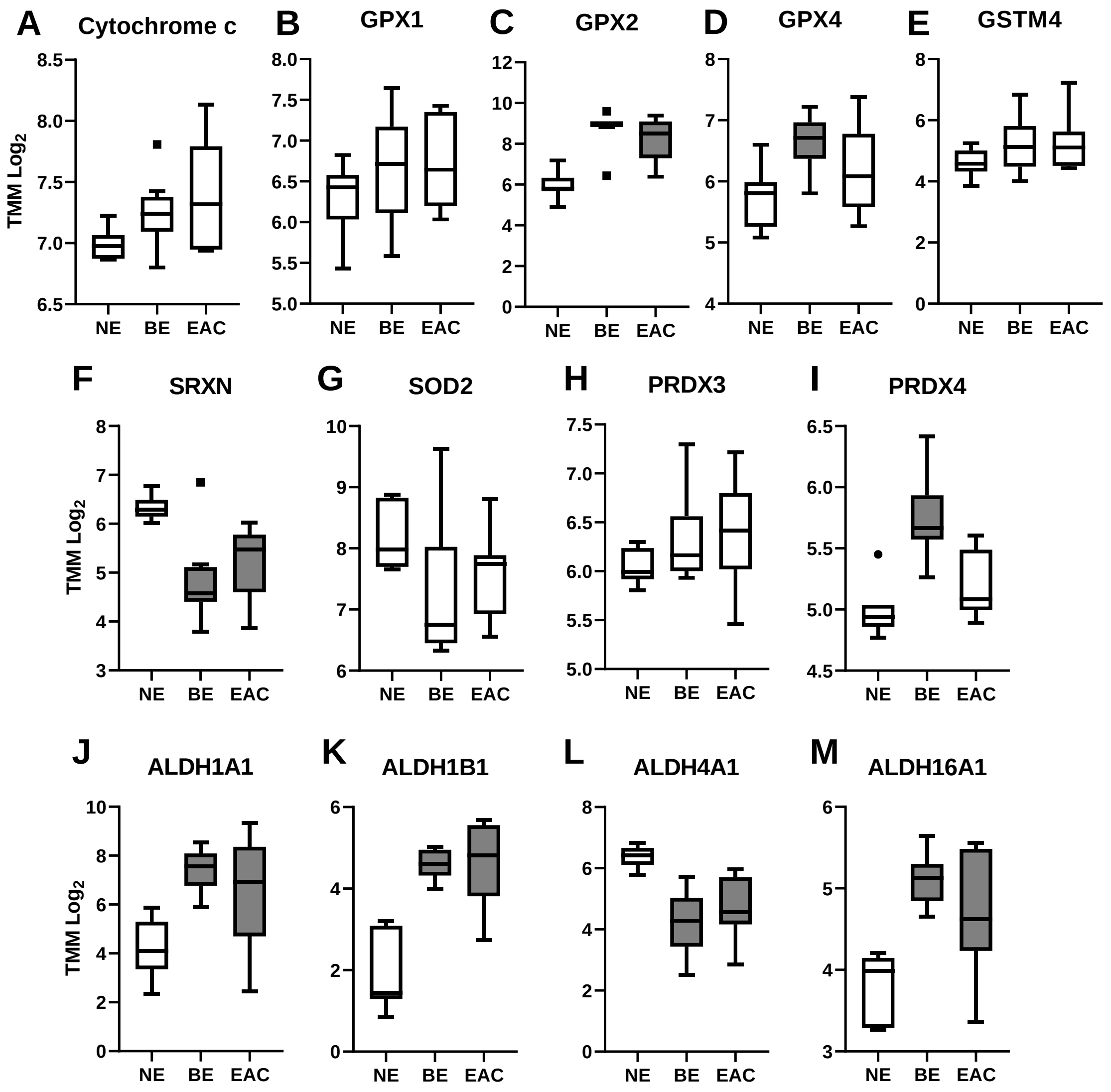

Figure S5. Patient tissue transcriptomic data for selected redox genes. TMM $\log_2$ expression for a range of redox gene transcripts in normal esophagus (NE, n=7), Barrett's Esophagus (BE, n=9) and esophageal adenocarcinoma (EAC, n=8). Shaded boxes indicate significance (P<0.05) by one way ANOVA compared to NE, overrepresentation p-values were adjusted using Benjamini-Hochberg correction.

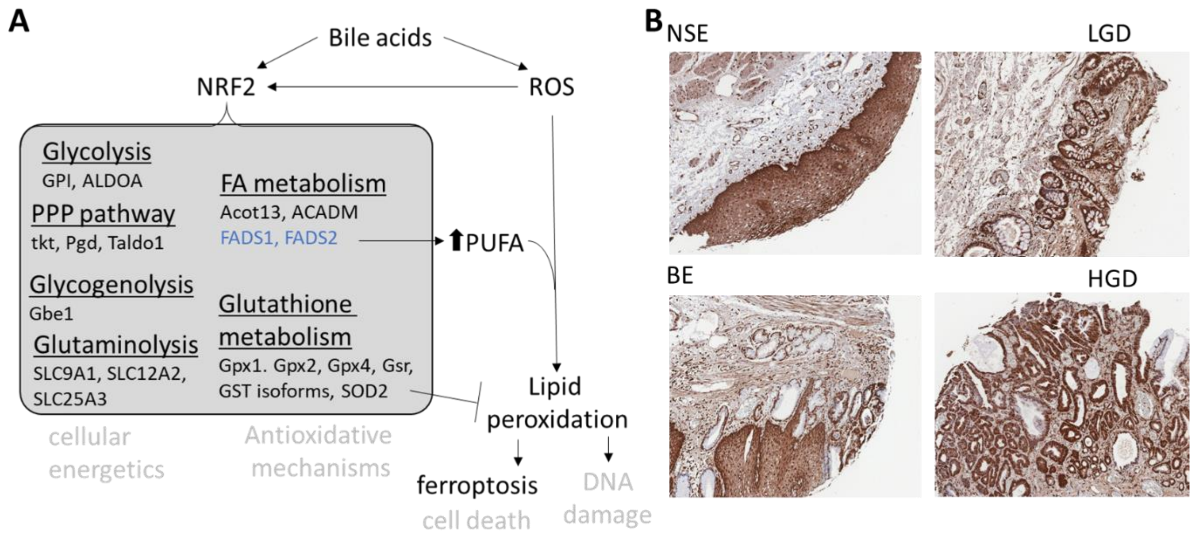

Figure S6. NRF2 is not altered in EAC progression. (A) Many of the pathways identified by proteomics (shown in black) or by protein immunohistochemistry and transcriptome data (blue) are controlled by NRF2, which can be activated by bile acids and ROS, and may lead to increased PUFA and lipid peroxidation. However, we observe no change in NRF2 staining during the progression from normal squamous epithelium (NSE), Barrett's esophagus (BE), low-grade dysplasia (LGD), or high-grade dysplasia (HGD) (B).

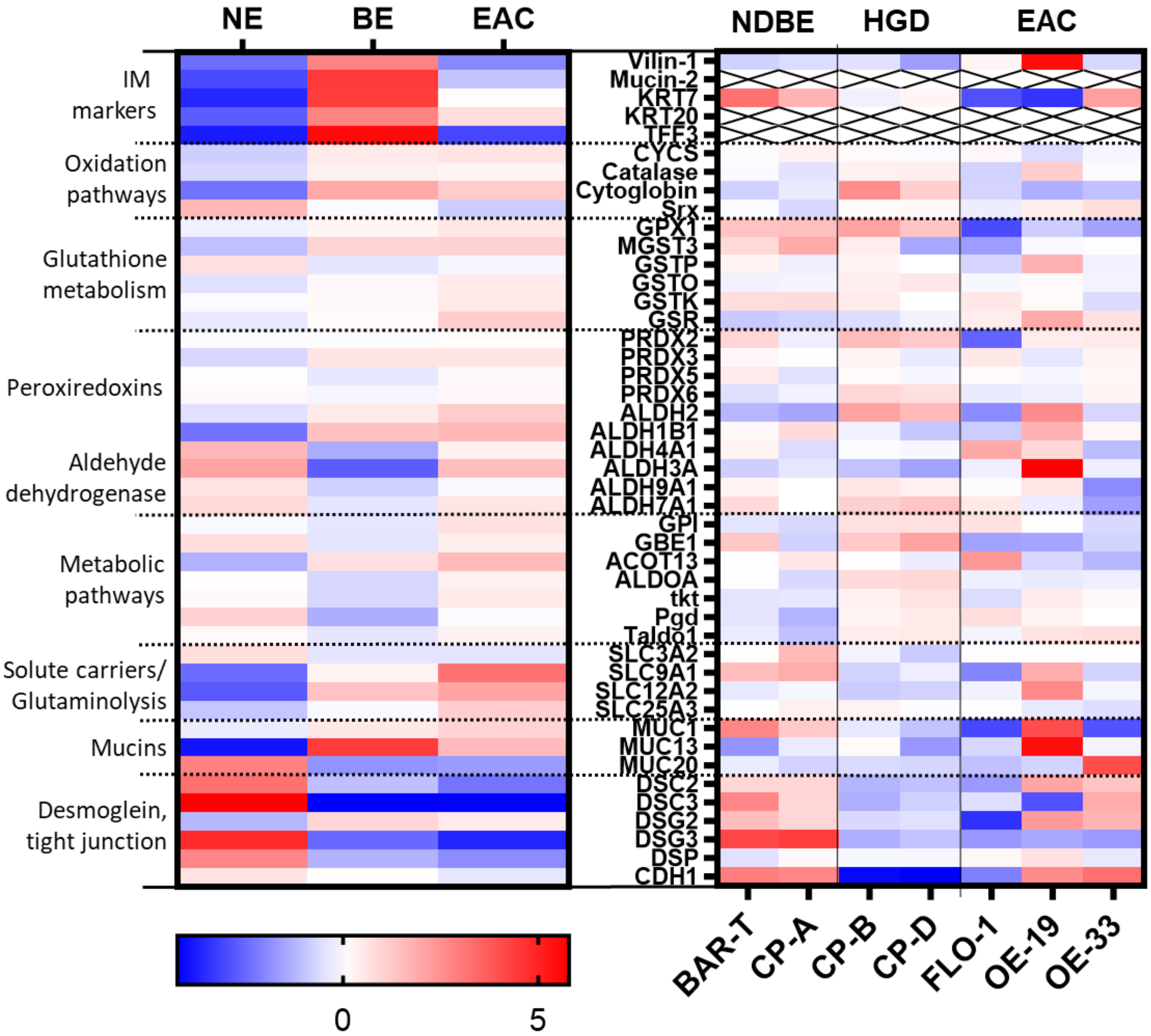

Figure S7. Comparison of human tissue and cell line proteome for BE and EAC. Mean-centered log2 abundance, focusing on markers of intestinal metaplasia, oxidation pathways, metabolic pathways, and esophageal defences such as mucins and desmosomes. Patterns observed in the human cohort (normal esophagus: NE, Barrett's esophagus: BE, and esophageal adenocarcinoma: EAC) were not replicated in the cell line analyses (NDBE: non-dysplastic BE, HGD: High-grade dysplasia).

SUPPLEMENTARY METHODS

Lipidomics: patient samples

The quadrupole was tuned to reference masses 112.99, 601.98 and 1333.97 in and the TOF component was tuned using reference masses 112.99, 302.00, 601.98, 1033.99, 1333.97 and 1633.95. The source capillary voltages were set to 3500 V and auto-recalibration using known reference masses (69.10, 112.99 and 1033.99 Da) was enabled. The reversed phase buffers used for negative ionization mode were the same as used in positive ionization, except for the addition of 10 mM ammonium acetate instead of the previously used ammonium formate and formic acid.

Lipids with a coefficient of variation (CV) > 30% among the quality control samples were removed from the dataset. For the untargeted lipidomics data, linear models of HPLC retention times were produced for the lipid classes PC, PE, LPC, LPE, PI, Cer, SM and TG. These models allowed the assessment of expected and measured retention times based on the lipid class, chain length and number of unsaturated bonds. Additionally, Kendrick mass defects (KMD) were calculated to ensure that the referenced KMD (RKMD) were consistent with the number of unsaturated bonds in the identified lipid molecule. Lipids for which the RKMD were not in agreement with the number of unsaturated bonds were excluded from the analyses.

Lipidomics: cell samples

Lipid extraction: Briefly, cell pellets were resuspended in 10µL of ice-cold milli-Q water before addition of 200µL ice-cold butanol/methanol (1:1) containing 50µg/mL antioxidant butylated hydroxytoluene (BHT) and 10mM ammonium formate. After vortexing, 10µL of a 1/10-diluted internal standard lipid mixture (SPLASH Lipidomix, Avanti #330707) was spiked into each sample to assess sample preparation and liquid chromatography retention time consistency across samples. The samples were incubated in a thermomixer for 1 hour at 4°C/850rpm before centrifugation for 15 minutes at 4°C/21,000*g*. The supernatant was removed and dried down in a vacuum concentrator for approx. 120 minutes. The resultant residue was resuspended in 50µL methanol/toluene (9:1) containing 100ng/mL 1-cyclohexyl-dodecanoic acid urea (CUDA). CUDA serves as an internal standard to monitor autosampler injection inconsistencies. Finally, samples were centrifuged for 5 minutes at 4°C/21,000*g* and the supernatant was transferred to a vial for analysis by LC/MS. In addition, a 5μL aliquot of each sample was combined to prepare a pooled QC sample to aid preliminary spectra analysis.

Targeted lipidomics was performed using method modified from Huynh et al [69] on an Agilent Technologies 1290 Infinity II LC System with a Zorbax Eclipse Plus C18 RRHD 2.1×100mm 1.8μm column, coupled to an Agilent 6470A Triple Quadrupole Mass Spectrometer via Jet Stream ionisation source. The sheath gas was set at 250°C at a flow rate of 10L/min, capillary voltage at 4750V, nebuliser at 20psi, and the column at 60°C. The mass spectrometer was operated in dynamic MRM mode. Ultra-performance liquid chromatography separation was achieved employing eluent A; acetonitrile/milliQ water/2-propanol (30:50:20, v/v), and eluent B; acetonitrile/milliQ water/2-propanol/ (9:1:90, v/v) with both eluents containing 10mM ammonium formate. Experiments were performed in biological triplicates.

Proteomics

Protein pellets were resuspended in 45 µL lysis buffer containing 10 mM TCEP, 40 mM 2CAA and 1% SDC in 100 mM TRIS. Samples were heated at 95°C for 5 min, followed disruption in a Bioruptor ultrasonicator (Diagenode, Liege, Belgium) (10 cycles of 30 sec vortex, 30 sec rest). After centrifugation for 10 min at 5000 × g, the supernatant was collected as tissue lysate. Protein concentration was determined by spotting 2 µL lysate on Millipore DirectDetect cards (Merck, Sydney, Australia) and dried before measurements. Lysis buffer was used as a blank, measurements were made using the AM2.q3 setting. Trypsin digestion was performed at 37°C with 20 µg sample in 24 µL lysis buffer, and 0.4 µg sequencing grade modified trypsin (Promega #V5111) in 180 µL trypsin resuspension buffer. After overnight incubation, samples were acidified using 10 µL of 10% TFA and centrifuged at 3,200 × g for 10 min to remove particulates. The supernatant (160 µL) was

collected and cleaned using Glygen 250 µL C-18 Velotips (Velo-C18-20µg). Solvents were removed by drying in a Savant Speedvac concentrator (Thermo Fisher Scientific, Brisbane, Australia) at 45°C. Dried peptides were stored at -20°C until use, then resuspended in 0.1% formic acid in MilliQ water prior to mass spectrometry analysis.

Trypsin digested samples were separated using the Thermo Easy-nLC 1000 with NanoViper separation column (#6041.5261) and Acclaim PepMap 100 NanoViper C18 trap column (#164705). A 180-minute gradient of buffer A (0.1% formic acid in MilliQ water) and buffer B (0.1% formic acid in acetonitrile) was run with a 180min gradient (Flow 250nl/min, Time 0min: 3%B, 3min: 6%B, 143min: 25%B, 163min: 40min, 164min 95%B, 179min 95%B, 180min: 3%B). Peptides were analyzed on a Thermo Orbitrap QE plus mass spectrometer. The Chromatography Peak width was set to 12 seconds. Dynamic exclusion was set to 10 ppm. A full MS / dd-MS2 (TopN) was run in positive polarity with an in-source collision-induced dissociation of 0 V. Default charge state was set to 2, microscans set to 1 and a resolution of 70,000 was used. The acquisition target was set to 3e6 with a maximum injection time of 100 ms. The scan range was 350 to 1400 m/z and the spectrum "profile" data type was used. The dd-MS2 / dd-SIM settings were set to a resolution of 17,500, automatic gain control target of 5e5, maximum injection time of 55 ms, loop count of 20, MSX count of 1 and TopN of 20. The isolation window was set to 1.2 m/z, isolation offset of 0 m/z, scan range from 200-2000 m/z and fixed first mass of 140 m/z. NCE was 29, spectrum data were saved as centroid. MaxQuant version 1.6.1.0 was used for feature extraction and protein identification with the human Swiss-Prot database containing 20,258 reviewed proteins (downloaded February 2018). The digestion enzyme was set to Trypsin/P with a maximum of two missed cleavages and carbamidomethyl as fixed modification. Variable modifications were set to oxidation and acetylation with a maximum number of five variable modifications per peptide. Default instrument settings for Orbitrap mass spectrometers were used. Match type was set to "Match from and to". Sequence data from Swiss-Prot was loaded and carbamidomethyl fixed modifications were allowed. Minimum peptide length was set to 5 and maximum mass to 4600 Da, peptide length for unspecified search was set between 6 and 25. For identification a score of 30 was required for modified peptides and at least one razor or unique peptide needs to be detected. The "match between runs" parameter was set to a match time window of 4 minutes and an alignment window of 25 minutes to recursively identify features between samples. For quantitation the label minimum ratio count was set to two, using only unique and razor peptides (modified and unmodified) with advanced ratio estimation. Large LFQ ratios were stabilized and advanced site intensities was selected.

Immunohistochemical staining

Sections were first affixed to positively-charged adhesive slides and air-dried overnight at 37°C before dewaxing and dehydration with descending graded alcohols and water. Sections were then incubated in Tris Buffered Saline (TBS) at pH 7.6 with 2% (FADS2) or 3% (FADS1, Nrf2) H_2O_2 for 10 minutes to block endogenous peroxidase activity. Sections were washed in three changes of water and transferred to Dako Epitrope Retrieval Buffer (FADS2: pH 9.0, 20min at 100°C heat retrieval. FADS1: pH6.0, 20min at 80°C. Nrf2: pH 6.0, 5min at 125°C). After cooling for 20 minutes, the sections were washed in TBS with 0.05% Tween (TBS_{TW}). Biocare Medical Background Sniper and 2% BSA were added for 15 minutes to inhibit non-specific antibody binding. Excess Sniper BSA was removed from the sections. FADS2 primary antibody (Abcam#ab232898) was diluted 1:25 in Da Vinci Green antibody diluent and applied overnight at room temperature. FADS1 antibody (Abcam#ab236672) was diluted 1:200, DEGS1 antibody (Abcam#ab167169) was diluted 1:200, and Nrf2 antibody (Abcam#ab137550) was diluted 1:700 in Da vinci Green and applied one hour at room temperature. Sections were washed three times in TBS_{TW} and Biocare Medical MACH1 Universal was applied for 20 minutes (30minutes for Nrf2). The sections were again washed in TBS_{TW} before signals were developed in NovaRed for 3 minutes, or DAB for 2 minutes (Nrf2). To remove excess chromogen, the sections were washed in water before the application of a light haematoxylin

counterstain. Finally, the sections were washed in water, dehydrated through ascending graded alcohols, cleared in xylene and mounted with DePeX.

Cell culture

Non-dysplastic BE and dysplastic esophageal cell lines CP-A, CP-B and CP-D were provided by Dr P. Rabinovitch (University of Washington, USA); BAR-T cells were provided by Dr Rhonda Souza (Baylor University Medical Center, TX). All non-dysplastic BE and dysplastic esophageal cells were cultured in Keratinocyte Serum Free Medium (KSFM) in 2.5% heat inactivated fetal bovine serum (HI FBS) (Gibco), supplemented with 0.1 µg/mL Epidermal Growth Factor (PeproTech) and 0.7 µg/mL of Bovine Pituitary Extract (Sigma). OE33 and FLO-1 were purchased from the European Collection of Authenticated Cell Cultures (Sigma). OE33 were cultured in Roswell Park Memorial Institute 1640 medium (RPMI) in 10% HI FBS, while FLO-1 were cultured in Dulbecco's modified Eagle's medium (DMEM) in 2.5% HI FBS. All cell lines were cultured in a 5% CO_2 incubator set to 37°C and underwent proteomic analysis. Cell-line identity homogeneity was confirmed through short tandem repeat (STR) profiling and mycoplasma testing was performed to exclude occult culture contamination.

Immunofluorescence

FLO-1 cells on coverslips were treated with 500nM SC26196 or vehicle (DMSO) for 48-hours before treatment with 1000µM bile acid cocktail (20 minutes in pH 4 media) or 1µM camptothecin (60 minutes in pH 7 media). Negative control cells were maintained in pH 7 media. Cells were washed, fixed in 4% PFA for 20 minutes, and incubated for 60 minutes in 2% BSA-PBS with 0.5% Triton X100 for 60 minutes to permeabilize and block non-specific antibody binding. Coverslips were incubated in a primary γH2AX antibody (anti-phospho-histone H2A.X (Ser139) (Sigma 05-636) 1:1000 in 2% BSA), for 60 minutes in a humidified chamber at 37°C. After washing in PBS, secondary antibody was applied (AlexaFluor488, 1:1000 in 2% BSA, 1 hr). After washing coverslips were incubated with DAPI for 5 minutes at room temperature before mounting. Immunofluorescence microscopy was performed using a Zeiss 780 NLO confocal microscope. Z-stack images were acquired with a 63X objective and maximum intensity projections were constructed in Zeiss Zen Black. Experiments were completed in biological quadruplicate and technical duplicate.
